# Supplementary figures and images for: Disruption of riboflavin biosynthesis in mycobacteria establishes riboflavin pathway intermediates as key precursors of MAIT cell agonists
Source: PLoS Pathog. 2025 Jul 1;21(7):e1012632. doi: 10.1371/journal.ppat.1012632 (PMC12240317; doi:10.1371/journal.ppat.1012632)

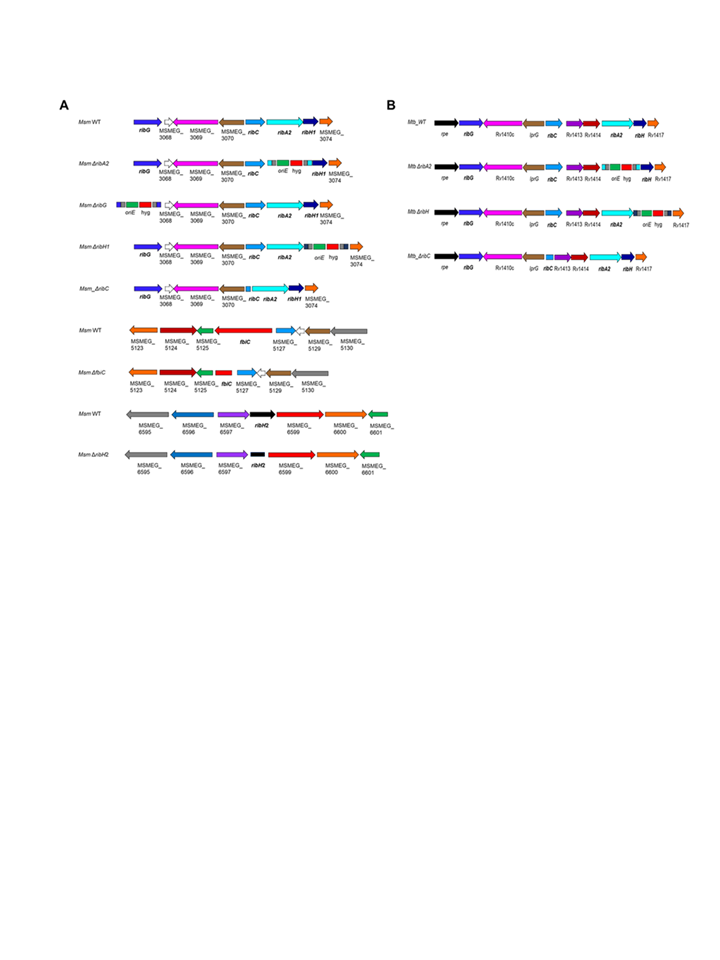

Supplement: S1 Fig — The mutants constructed by ORBIT carry a vector sequence which included a hyg resistance marker whereas the allelic exchange mutants are unmarked. (TIF) [file ppat.1012632.s001.TIF]

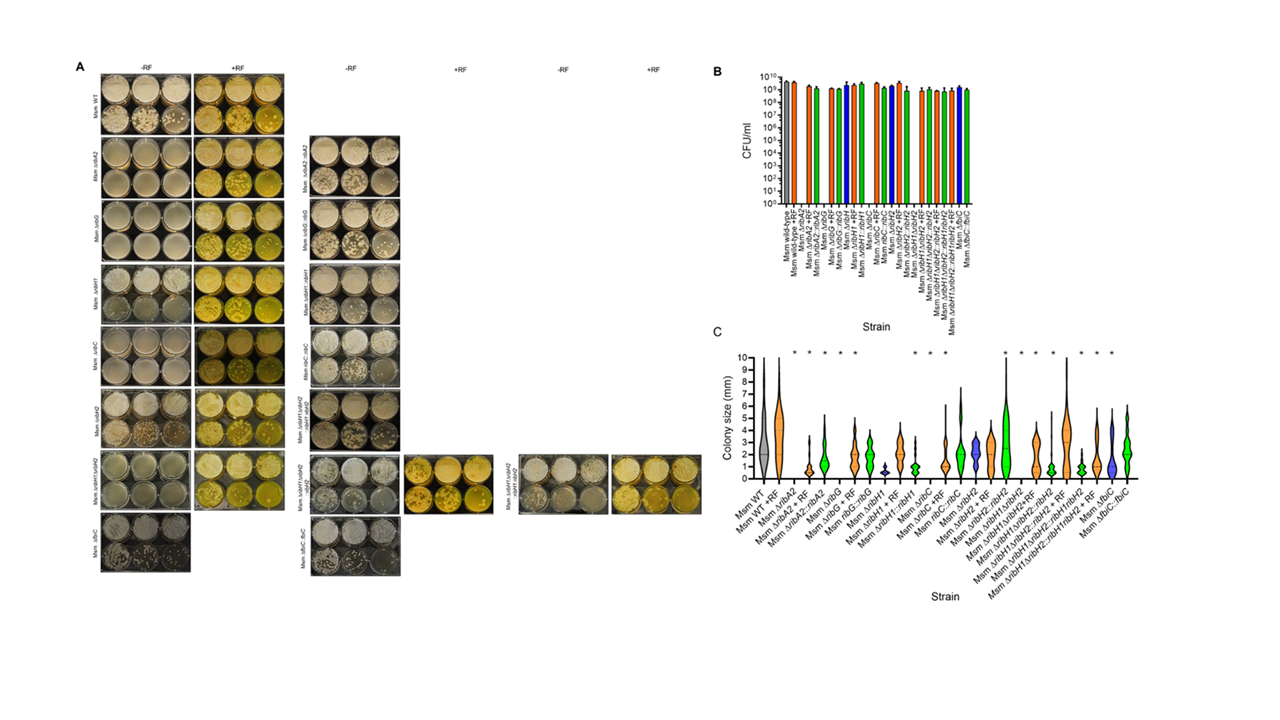

Supplement: S2 Fig — Msm cultures were diluted 10-fold, spread onto 6-well plates and incubated for 4 days (A) and CFUs enumerated. (B). Msm ΔribA2::ribA2, ΔribH1::ribH1, ΔribH2ΔribH1::ribH2 and ΔribH2ΔribH1::ribH2 ribH1 plates were incubated for an additional day as colonies were too small to count on day 4. The colony sizes of all emerging colonies were measured (C). Riboflavin supplement was used at a concentration of 83 μM for culturing Msm. Error bars represent standard deviation from two biological replicates. Statistical comparisons against wild-type were performed using a one-way ANOVA and Kruskal-Wallis comparison test whereby statistical significance is represented by p < 0.05, shown by an asterisk. (TIF) [file ppat.1012632.s002.tif]

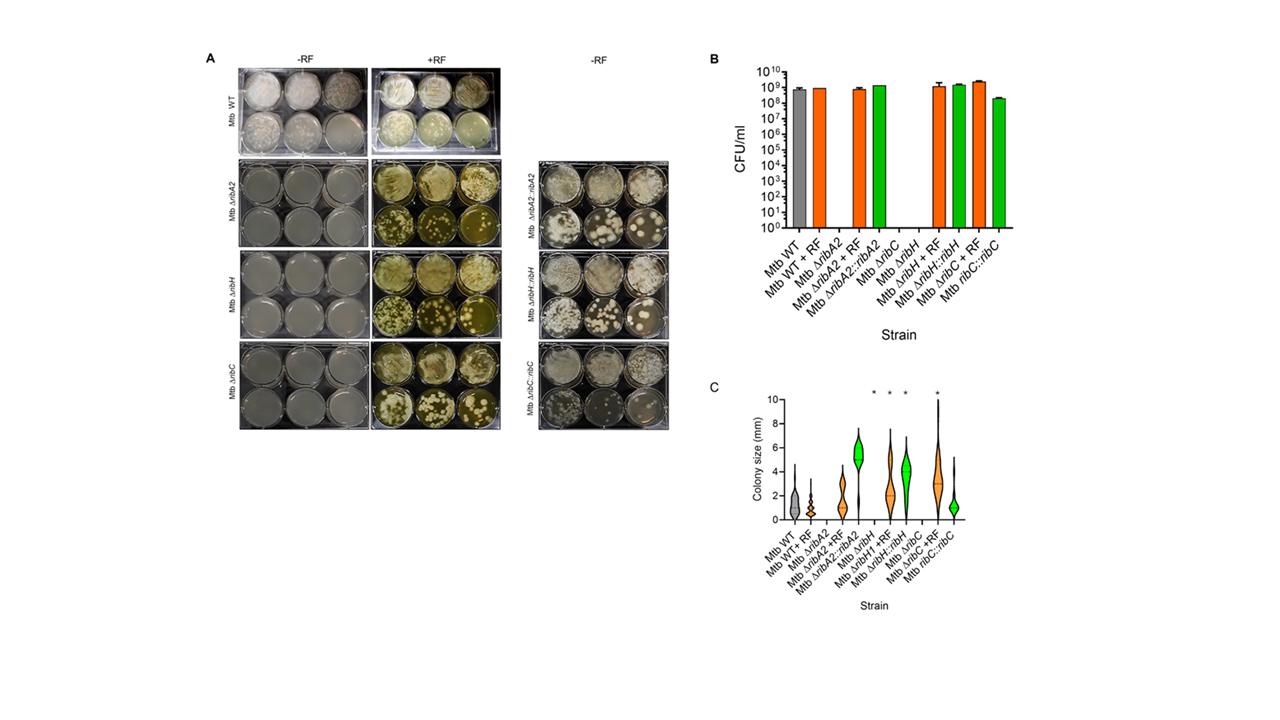

Supplement: S3 Fig — Mtb cultures were diluted 10-fold, spread onto 6-well plates and incubated for 4 weeks (A) and CFUs enumerated (B). The colony sizes of all emerging colonies were measured (C). Riboflavin supplement was used at a concentration of 21 μM for Mtb. Error bars represent standard deviation from two biological replicates. Statistical comparisons, against wild-type were performed using a one-way ANOVA and Kruskal-Wallis comparison test whereby statistical significance is represented by p < 0.05, shown by an asterisk. (TIF) [file ppat.1012632.s003.tif]

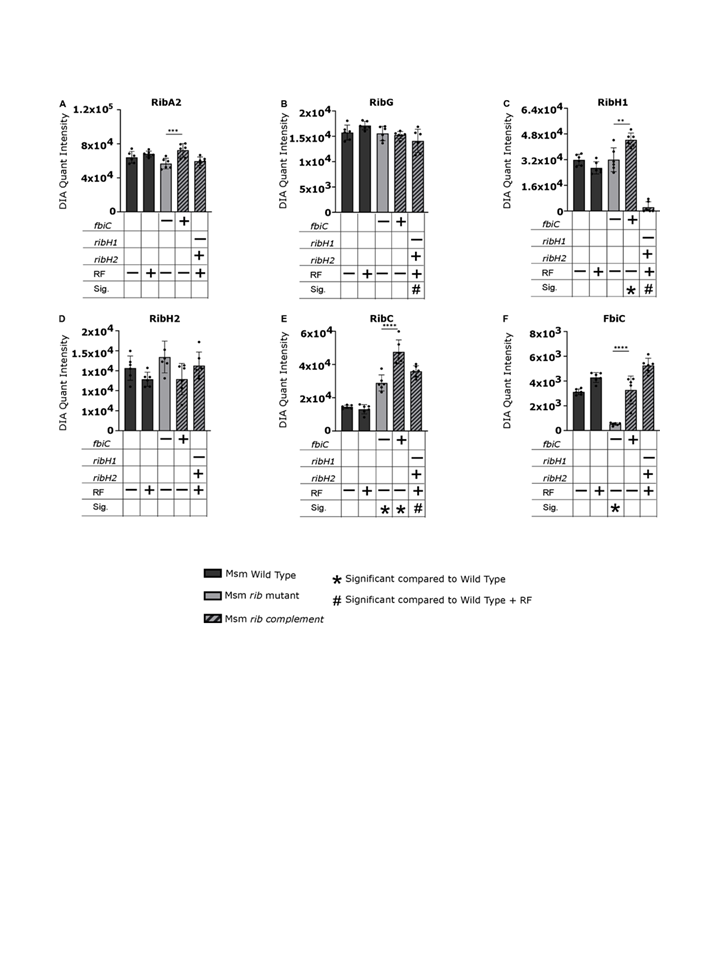

Supplement: S4 Fig — Protein abundance was measured using DIA proteomics (n = 3 biological replicates, 2 technical replicates). Data are shown as mean quantification ± SEM. Statistical comparisons were performed using a one-way ANOVA and Sidak’s multiple comparison test whereby statistical significance is represented by p < 0.05, p < 0.001, p < 0.0005 p < 0.0001, shown by *, **, ***, **** respectively. Only statistically significant relationships are shown. RF, riboflavin. Sig., significance in comparison to wild type (+/- RF) is shown as symbols as described in legend (p < 0.05). Wild type ± RF shown in this figure are the same as those in Figs 3 and 4. (TIF) [file ppat.1012632.s004.tif]

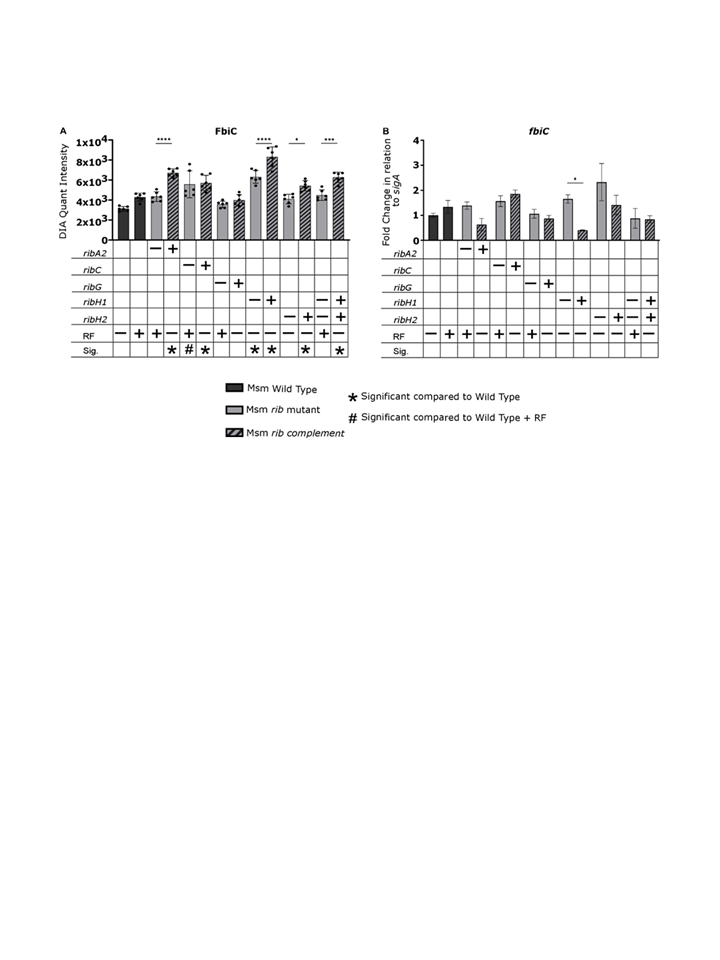

Supplement: S5 Fig — (A) Protein abundance was measured using DIA proteomics (n = 2 biological replicates, 2 technical replicates). Data are shown as mean quantification ± SEM. (B) Fold changes in sigA-normalized transcript levels relative to wildtype. Transcript levels of the target genes were normalized to the housekeeping gene sigA (an essential housekeeping gene which is stably expressed) and scaled to the average of wildtype Msm to calculate the ΔΔCt and determine the fold difference in gene expression (n = 3 biological replicates, 2 technical replicates). Statistical comparisons were performed using a one-way ANOVA and Sidak’s multiple comparison test whereby statistical significance is represented by p < 0.05, p < 0.001, p < 0.0005 p < 0.0001, shown by *, **, ***, **** respectively. Only statistically significant relationships are shown. RF, riboflavin. Sig., significance in comparison to wild type (+/- RF) is shown as symbols as described in legend (p < 0.05). (TIF) [file ppat.1012632.s005.tif]

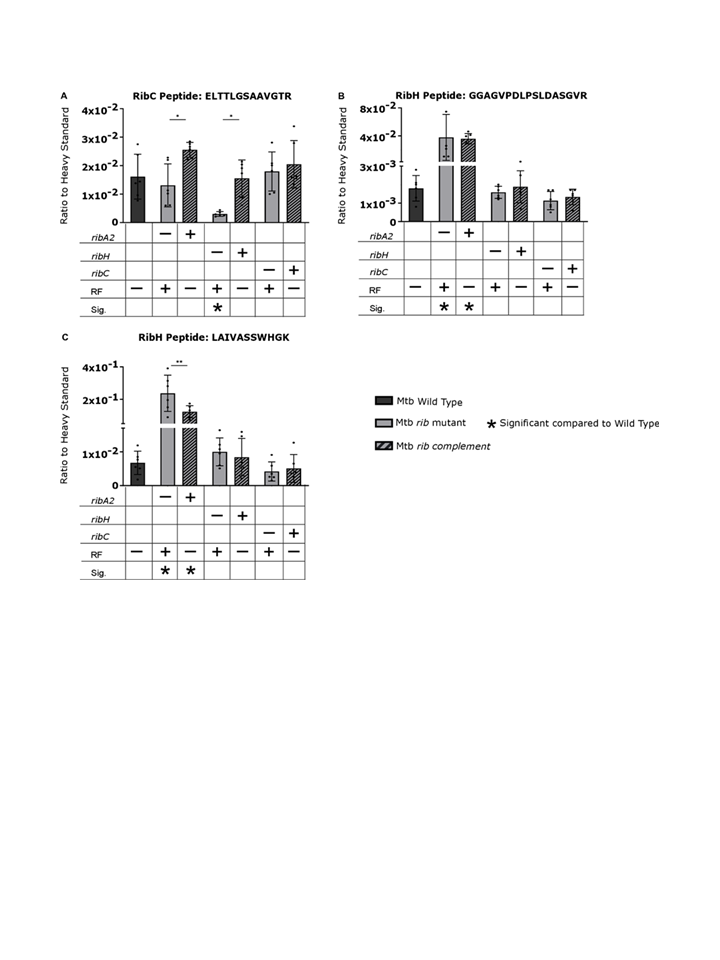

Supplement: S6 Fig — Stable isotope-labeled standard (SIS) peptides were selected for RibC and RibH for targeted quantification of proteins using LC-MS/MS. Ratio to heavy standard represents protein abundance of RibC (A), and RibH (B and C) in the different strains of Mtb. Data are shown as mean quantification ± SEM of three biological replicates. Statistical comparisons were performed using a one-way ANOVA and Sidak’s multiple comparison test whereby statistical significance is represented by p < 0.05, p < 0.001, p < 0.0005 p < 0.0001, shown by *, **, ***, **** respectively. Only statistically significant relationships are shown. RF, riboflavin. Sig., significance in comparison to wild type (+/- RF) is shown as symbols as described in legend (p < 0.05). (TIF) [file ppat.1012632.s006.tif]

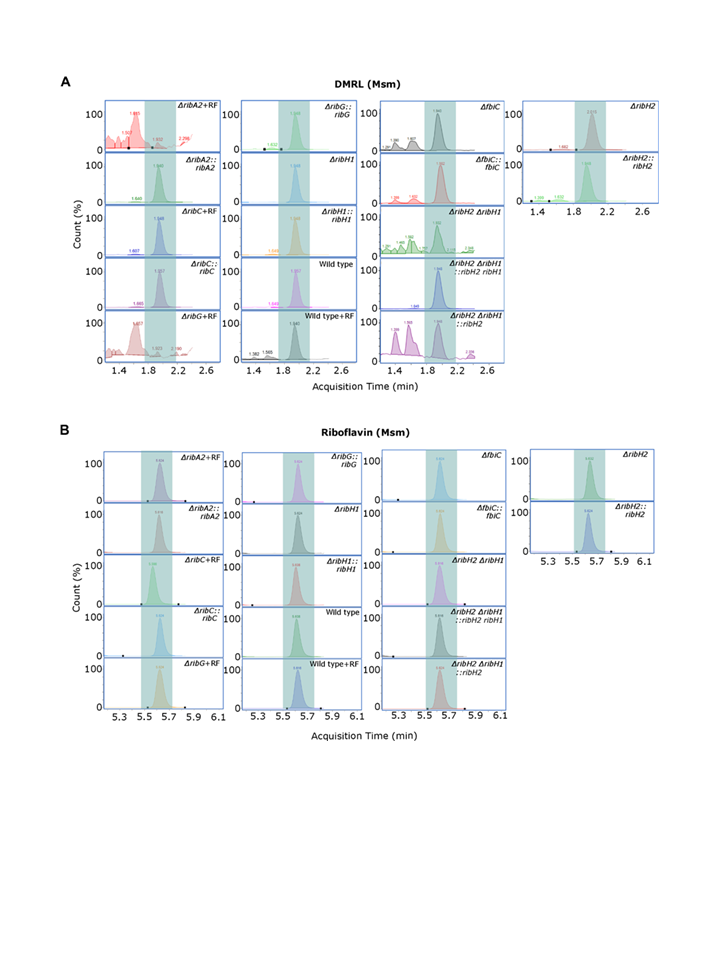

Supplement: S7 Fig — Representative chromatograms depicting the most intense multiple reaction monitoring (MRM) transitions for DMRL (A, m/z 327.1 → 193) and RF (B, m/z 377.2 → 243.1) in Msm strains. Each metabolite was monitored using its most intense transition ion, along with the next two most intense transition ions, to confirm specificity. (TIF) [file ppat.1012632.s007.tif]

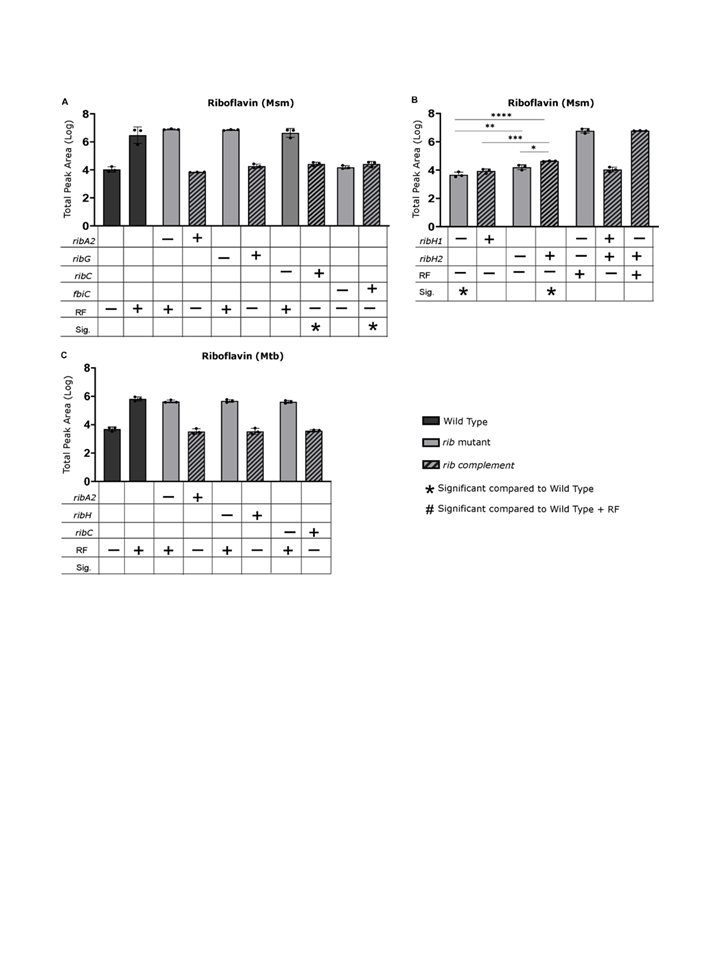

Supplement: S8 Fig — Levels of intracellular riboflavin were quantified in Msm (A and B) and Mtb (C) using MRM. Data are shown as mean quantification ± SEM for three biological replicates. Quantification was carried out using chromatographic peak area of the most intense transition ion (m/z 377.2 → 243.1) of RF. Statistical comparisons were performed using a one-way ANOVA and Sidak’s multiple comparison test whereby statistical significance is represented by p < 0.05, p < 0.001, p < 0.0005 p < 0.0001, shown by *, **, ***, **** respectively. Only statistically significant relationships are shown. RF, riboflavin. Sig., significance in comparison to wild type (+/- RF) is shown as symbols as described in legend (p < 0.05). Statistical comparison was done only between samples not spiked with exogenous riboflavin. (TIF) [file ppat.1012632.s008.tif]

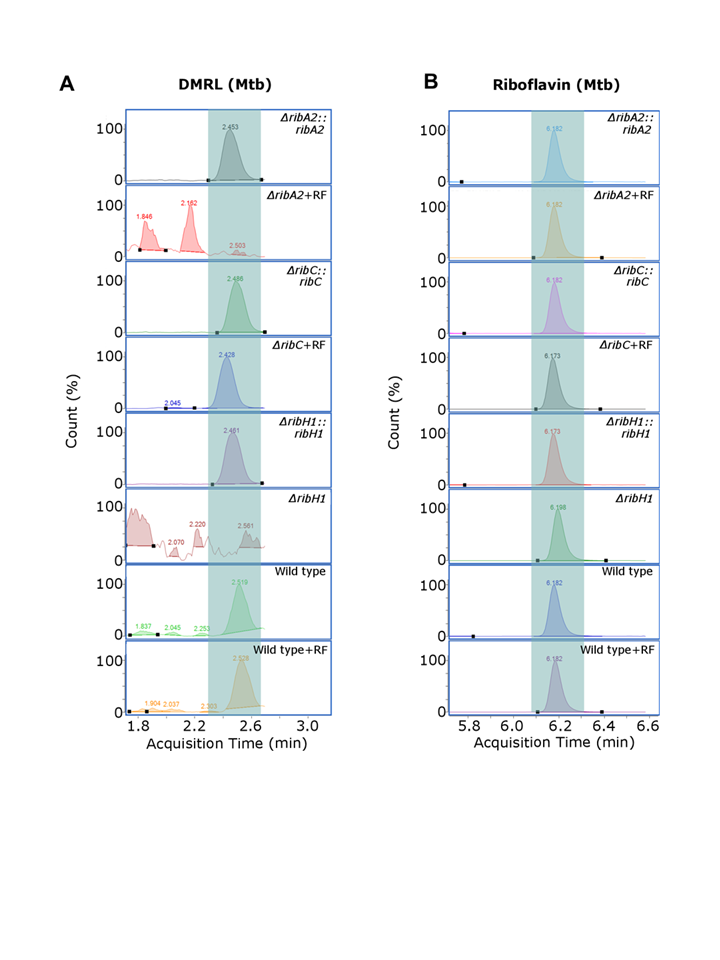

Supplement: S9 Fig — Representative chromatograms depicting the most intense multiple reaction monitoring (MRM) transitions for DMRL (A, m/z 327.1 → 193) and riboflavin (RF) (B, m/z 377.2 → 243.1) in Mtb strains. Each metabolite was monitored using its most intense transition ion, along with the next two most intense transition ions, to confirm specificity. (TIF) [file ppat.1012632.s009.tif]

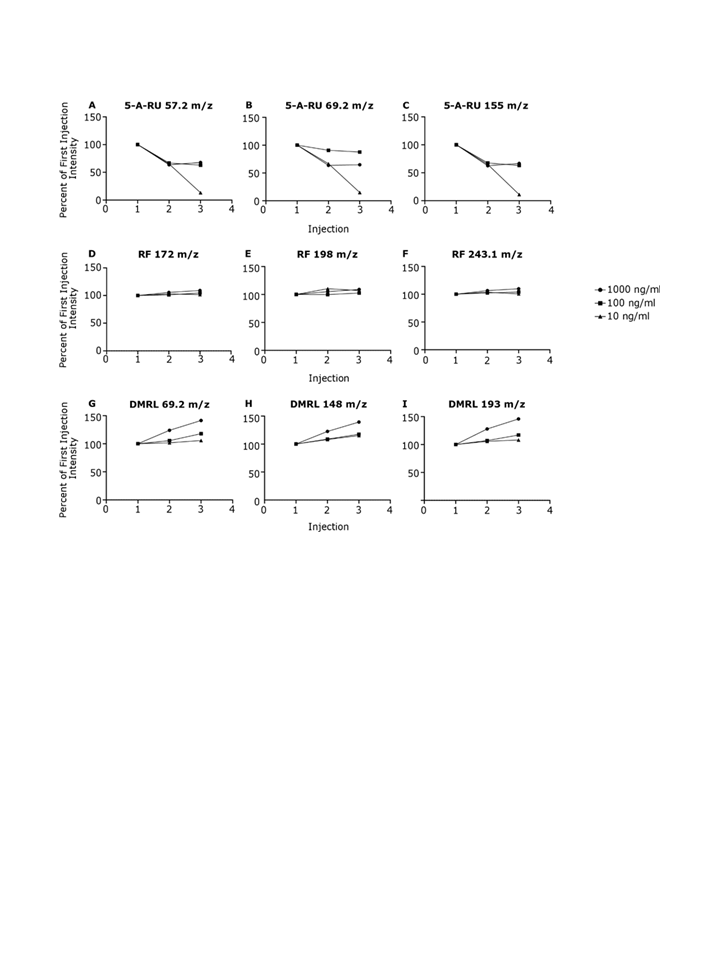

Supplement: S10 Fig — Stability of 5-A-RU (A, B, and C), riboflavin (D, E, and F) and DMRL (G, H, and I) were monitored using the three most intense transition ions over three injections with samples kept at 5°C with ≈ 11 minutes between injections. Data are shown as percentage of chromatographic peak area of initial injection. (TIF) [file ppat.1012632.s010.tif]

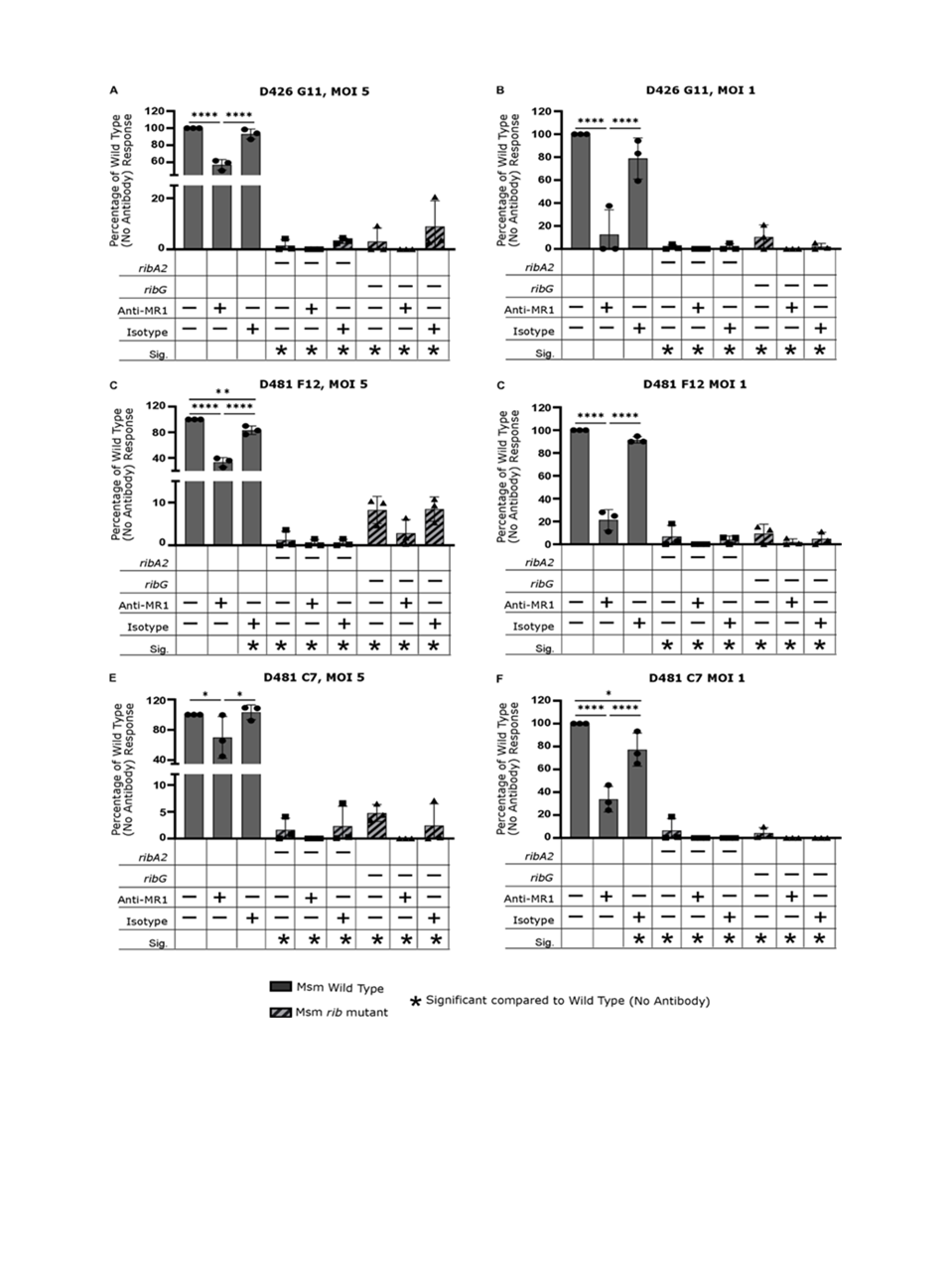

Supplement: S11 Fig — MR1T cell clone (1e4) IFN-γ response to dendritic cells (1e4) incubated with Msm wildtype, ΔribA2, or ΔribG at a MOI of 5 or MOI of 1 is inhibited by anti-MR1 antibody blockade (aMR1 or isotype, 2µg/ml). Response was normalized to wildtype Msm no antibody condition. Data are representative of n = 3 independent experiments. (A) D426 G11 at MOI 5, (B) D426 G11 at MOI 1, (C) D481 F12 at MOI 5, (D) D481 F12 at MOI 1, (E) D481 C7 at MOI 5, (F) D481 C7 at MOI 1. Statistical comparisons were performed using a one-way ANOVA and Sidak’s multiple comparison test whereby statistical significance is represented by p < 0.05, p < 0.001, p < 0.0005 p < 0.0001, shown by *, **, ***, **** respectively. (TIF) [file ppat.1012632.s011.tif]

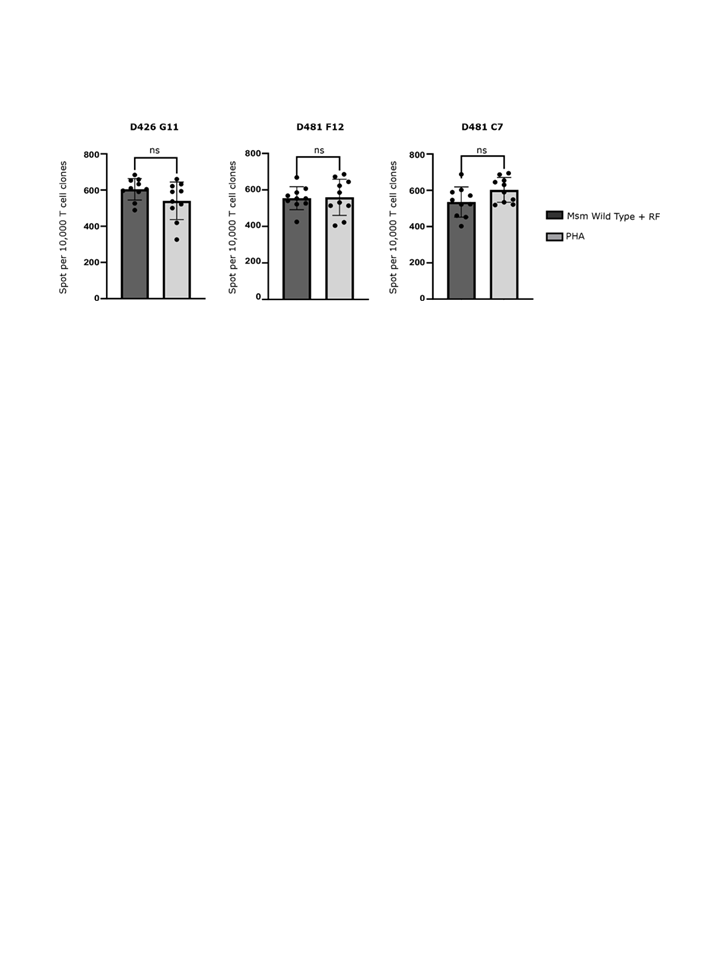

Supplement: S12 Fig — Shown are the spot counts from MR1T cell clones (1e4) IFN-γ response to DCs (1e4) for the wildtype Msm (MOI 10) and PHA (10ug/ml) conditions for every experiment included in Fig 7. Statistical comparisons were performed using a one-way ANOVA and Sidak’s multiple comparison test whereby statistical significance is represented by p < 0.05, p < 0.001, p < 0.0005 p < 0.0001, shown by *, **, ***, **** respectively. (TIF) [file ppat.1012632.s012.tif]

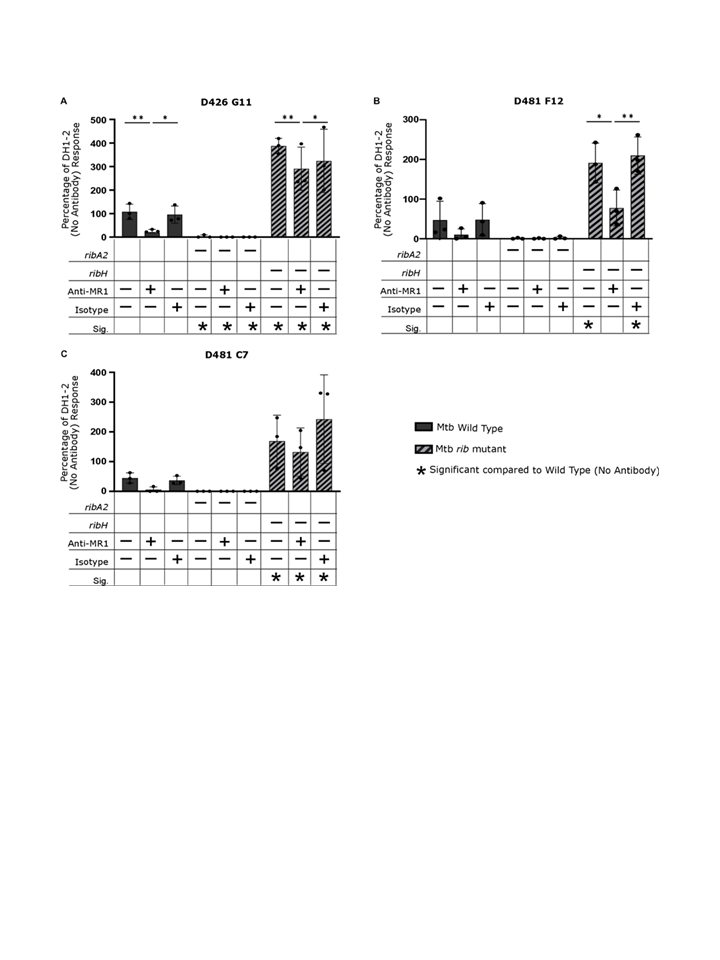

Supplement: S13 Fig — MR1T cell clone (1e4) IFN-γ response to dendritic cells (1e4) incubated with Mtb wildtype, ΔribA2 or ΔribH at a MOI of 5 is inhibited by anti-MR1 antibody blockade (aMR1 or isotype, 2µg/ml). Response was normalized to wildtype Mtb no antibody condition. Data are representative of n = 3 independent experiments. Statistical comparisons were performed using a one-way ANOVA and Sidak’s multiple comparison test whereby statistical significance is represented by p < 0.05, p < 0.001, p < 0.0005 p < 0.0001, shown by *, **, ***, **** respectively. (TIF) [file ppat.1012632.s013.tif]
