# Supplementary material for: Disruption of riboflavin biosynthesis in mycobacteria establishes riboflavin pathway intermediates as key precursors of MAIT cell agonists
Source: PLoS Pathog. 2025 Jul 1;21(7):e1012632. doi: 10.1371/journal.ppat.1012632 (PMC12240317; doi:10.1371/journal.ppat.1012632)
Supplement: S1 Table — (DOCX) [file ppat.1012632.s014.docx]

**S1 Table.** TCR sequence of MR1T cell clones

| **Clone name** | **TRAV** | **CDR3a** | **TRAJ** | **TRBV** | **CDR3b** | **TRBJ** |
| --- | --- | --- | --- | --- | --- | --- |
| D426 G11 | 1-2 | CAVRDSNYQLIW | 3 | 6-4 | CASSDSGESGTEAFF | 1-1 |
| D481 C7 | 1-2 | CAVSLQDYKLSF | 33 | 20-1 | CASSPSGGDYNEQF | 2-7 |
| D481 F12 | 1-2 | CAVRDSDYKLSF | 20 | 6-4 | CASSQIAGGQQETQY | 2-1 |
