# Supplementary material for: Disruption of riboflavin biosynthesis in mycobacteria establishes riboflavin pathway intermediates as key precursors of MAIT cell agonists
Source: PLoS Pathog. 2025 Jul 1;21(7):e1012632. doi: 10.1371/journal.ppat.1012632 (PMC12240317; doi:10.1371/journal.ppat.1012632)
Supplement: S2 Table — (DOCX) [file ppat.1012632.s015.docx]

**S2 Table.** Plasmids used in this study

| **Plasmid** | **Features** | **Reference** |
| --- | --- | --- |
| pLJR962 | ATc-inducible (P_tet_) *Streptococcus* *thermophilius* (Sth) dCas9 and P_tet_ sgRNA, integrating at the L5 locus in Msm; Kan^R^ | [1] |
| p2NIL | *E. coli* cloning vector; Kan^R^ | [2] |
| pGOAL17 | Plasmid carrying *lacZ* and *sacB* as a *Pac*I cassette; ampicillin resistant; Amp^R^ | [2] |
| pGOAL19 | Plasmid carrying *lacZ, sacB* and hyg as a *Pac*I cassette; ampicillin resistant; Amp^R^ | [2] |
| pMV306H | *E. coli*-Mycobacterium promoter-less L5 site integrating shuttle vector, derivative of pMV306; hygromycin resistant; Hyg^R^ | Helena Boshoff |
| pL5 | *E. coli*-Mycobacterium vector with L5 integrating site and P*_symc_* promoter, co-electroporated with pBS-Int; Kan^R^ | [3] |
| pBS-Int | pBluescript*,* L5 integrase; Amp^R^ | [3] |
| pTweetyG | *E. coli*-Mycobacterium promoter-less Tweety site integrating shuttle vector; Gent^R^ | [4] |
| pKM461 | Plasmid expresses P_tet_ Che9c phage RecT annealase and P_tet_ Bxb1 phage integrase; *oriE*, *oriM*, *sacB* ; Kan^R^, Tet^R^ | [5] |
| pKM464 | Non-replicating vector carrying *attB*; Hyg^R^ | [5] |
| pTwG_Msm_*ribA2* | Derivative of pTweetyG carrying the *ribA2* ORF and 321 bp upstream of the gene and 90 bp downstream; Gent^R^ | This study |
| pTwG_Msm_*ribG* | Derivative of pTweetyG carrying the *ribG* ORF and 279 bp upstream of the gene and 75 bp downstream; Gent^R^ | This study |
| pΔ_Msm_*ribC* | Derivative of p2NIL-pGOAL17 carrying a fragment containing 1972 bp upstream of *ribC* and 36 bp into *ribC* and another fragment containing 48 bp of the *ribC* terminus and 1757 bp downstream of *ribC;* Kan^R^ | This study |
| pMV_Msm_*ribC* | Derivative of pMV306H carrying the *ribC* ORF and 300 bp upstream of the gene*;* Hyg^R^ | This study |
| pTwG_Msm_*ribH1* | Derivative of pTweetyG carrying the *ribH1* ORF and 291 bp upstream of the gene and 67 bp downstream; Gent^R^ | This study |
| pΔ_Msm_*ribH2* | Derivative of p2NIL-pGOAL19 carrying a fragment containing 925 bp upstream of, and 108 bp of the 5’-terminal region of *ribH2* and another fragment containing 69 bp of the 3’-terminal region of ribH2 and 1017 bp downstream of *ribH2*; Kan^R^ and Hyg^R^ | This study |
| pL5_*ribH2* | Derivative of pL5 carrying the *ribH*2 ORF and 318 bp upstream of the gene and 84 bp downstream, P*_symc_* promoter was removed; Kan^R^ | This study |
| pΔ_Msm_*fbiC* | Derivative of p2NIL-pGOAL19 carrying a fragment containing 1000 bp upstream of *fbiC* and 27 bp into *fbiC* and another fragment containing 183 bp of the *fbiC* terminus and 963 bp downstream of *fbiC;* Kan^R^ and Hyg^R^ | This study |
| pTwG_Msm_*fbiC* | Derivative of pTweetyG carrying the *fbiC* ORF and 310 bp upstream of the gene and 20 bp downstream; Gent^R^ | This study |
| pTwG_Mtb_*ribA2* | Derivative of pTweetyG carrying the *ribA2* ORF and 333 bp upstream of the gene and 90 bp downstream; Gent^R^ | This study |
| pΔ_Mtb_*ribC* | Derivative of p2NIL-pGOAL17 carrying a fragment containing 1565 bp upstream of *ribC* and 99 bp into *ribC* and another fragment containing 48 bp of the *ribC* terminus and 1591 bp downstream of *ribC;* Kan^R^ | This study |
| pMV_Mtb_*ribC* | Derivative of pMV306H carrying the *ribC* *ORF* and 422 bp upstream of the gene and 50 bp downstream*;* Hyg^R^ | This study |
| pTwG_Mtb_*ribH* | Derivative of pTweetyG carrying the *ribH* ORF and 344 bp upstream of *ribH* and 57 bp downstream; Gent^R^ | This study |

**References**

1. Rock JM, Hopkins FF, Chavez A, Diallo M, Chase MR, Gerrick ER, et al. Programmable transcriptional repression in mycobacteria using an orthogonal CRISPR interference platform. Nat Microbiol. 2017;2:16274.

2. Parish T, Stoker NG. Use of a flexible cassette method to generate a double unmarked Mycobacterium tuberculosis tlyA plcABC mutant by gene replacement. Microbiology (Reading). 2000;146 ( Pt 8):1969-75.

3. Kolbe K, Bell AC, Prosser GA, Assmann M, Yang HJ, Forbes HE, et al. Development and Optimization of Chromosomally-Integrated Fluorescent Mycobacterium tuberculosis Reporter Constructs. Front Microbiol. 2020;11:591866.

4. Pham TT, Jacobs-Sera D, Pedulla ML, Hendrix RW, Hatfull GF. Comparative genomic analysis of mycobacteriophage Tweety: evolutionary insights and construction of compatible site-specific integration vectors for mycobacteria. Microbiology (Reading). 2007;153(Pt 8):2711-23.

5. Murphy KC, Nelson SJ, Nambi S, Papavinasasundaram K, Baer CE, Sassetti CM. ORBIT: a New Paradigm for Genetic Engineering of Mycobacterial Chromosomes. mBio. 2018;9(6).
