# Supplementary material for: Disruption of riboflavin biosynthesis in mycobacteria establishes riboflavin pathway intermediates as key precursors of MAIT cell agonists
Source: PLoS Pathog. 2025 Jul 1;21(7):e1012632. doi: 10.1371/journal.ppat.1012632 (PMC12240317; doi:10.1371/journal.ppat.1012632)
Supplement: S3 Table — (DOCX) [file ppat.1012632.s016.docx]

**S3 Table.** Strains used in this study

| **Strain** | **Description** | **Reference** |
| --- | --- | --- |
| *Escherichia coli* DH5α | F^–^ φ80*lac*ZΔM15 Δ(*lac*ZYA-*arg*F)U169 *rec*A1 *end*A1 *hsd*R17(r_K_^–^, m_K_^+^) *pho*A *sup*E44 λ^–^ *thiA*-1 *gyr*A96 *rel*A1 | New England Biolabs |
| *M. smegmatis* mc^2^ 155 (Msm_WT) | High frequency transformation mutant of Msmc26 | [1] |
| Msm Δ*ribA2* | Riboflavin auxotroph in frame deletion mutant lacking internal region in *ribA2* generated using construct pKM464 and directed oligo; Hyg^R^ marked mutant generated using ORBIT | This study |
| Msm Δ*ribA2*::*ribA2* | Complemented mutant generated by introducing construct pTwG_Msm_*ribA2* in strain Msm Δ*ribA2;* Gent^R^ | This study |
| Msm Δ*ribG* | Riboflavin auxotroph in frame deletion mutant lacking internal region in *ribG* generated using construct pKM464 and directed oligo; Hyg^R^ marked mutant generated using ORBIT | This study |
| Msm Δ*ribG*::*ribG* | Complemented mutant generated by introducing construct pTwG_Msm_*ribG* in strain Msm Δ*ribG;* Gent^R^ | This study |
| Msm Δ*ribH1* | In frame deletion mutant lacking internal region in *ribH1* generated using construct pKM464 and directed oligo; Hyg^R^ marked mutant generated using ORBIT. This strain is not a RF auxotroph. | This study |
| Msm Δ*ribH1*::*ribH1* | Complemented mutant generated by introducing construct pTwG_Msm_*ribH1* in strain Msm Δ*ribH1;* Gent^R^ | This study |
| Msm Δ*ribC* | Riboflavin auxotroph in frame deletion mutant lacking internal region in *ribC* generated using construct pΔ_Msm_*ribC*; unmarked mutant generated using allelic exchange | This study |
| Msm Δ*ribC*::*ribC* | Complemented mutant generated by introducing construct pMV_Msm_*ribC* in strain Msm Δ*ribC;* Hyg^R^ | This study |
| Msm Δ*ribH2* | In frame deletion mutant lacking internal region in *ribH2* generated using construct pΔ_*ribH2*; unmarked mutant generated using allelic exchange | This study |
| Msm Δ*ribH2*::*ribH2* | Complemented mutant generated by introducing construct pL5_*ribH*2 in strain Msm Δ*ribH2* Kan^R^ | This study |
| Msm Δ*ribH2*Δ*ribH1* | In frame deletion mutant lacking internal region in *ribH1* generated using construct pKM464 and directed oligo and in frame deletion mutant lacking internal region in *ribH2* generated using construct pΔ_*ribH2*, generated using allelic exchange; Hyg^R^ | This study |
| Msm Δ*ribH2*Δ*ribH1*:: *ribH2* | Complemented mutant generated by introducing construct pL5_*ribH2* in strain Msm Δ*ribH1*Δ*ribH2*; Kan^R^ | This study |
| Msm Δ*ribH2*Δ*ribH1*:: *ribH2 ribH1* | Complemented mutant generated by introducing constructs pTwG_Msm_*ribH1* and pL5_*ribH2* in strain Msm Δ*ribH1*Δ*ribH2*; Gent^R^ and Kan^R^ | This study |
| Msm Δ*fbiC* | In frame deletion mutant lacking internal region in *fbiC* generated using construct pΔ_Msm_*fbiC*; unmarked mutant generated using allelic exchange | This study |
| Msm Δ*fbiC*::*fbiC* | Complemented mutant generated by introducing construct pTwG_Msm_*fbiC* in strain Msm *ΔfbiC* | This study |
| *M. tuberculosis* H37RvMA (Mtb WT) | Mtb H37Rv (ATCC 27294) virulent laboratory strain | [2] |
| Mtb pKM461 | Strain generated by introducing pKM461 into Mtb WT; Kan^R^ | This study |
| Mtb Δ*ribA2* | Riboflavin auxotroph in frame deletion mutant lacking internal region in *ribA2* generated using construct pKM464 and directed oligo; Hyg^R^ marked mutant generated using ORBIT | This study |
| Mtb Δ*ribA2*::*ribA2* | Complemented mutant generated by introducing construct pTwG_Mtb_*ribA2* in strain Mtb Δ*ribA2;* Gent^R^ | This study |
| Mtb Δ*ribH* | Riboflavin auxotroph in frame deletion mutant lacking internal region in *ribH* generated using construct pKM464 and directed oligo; Hyg^R^ marked mutant generated using ORBIT | This study |
| Mtb Δ*ribH*::*ribH* | Complemented mutant generated by introducing construct pTwG_Mtb_*ribH* in strain Mtb Δ*ribH;* Gent^R^ | This study |
| Mtb Δ*ribC* | Riboflavin auxotroph in frame deletion mutant lacking internal region in *ribC* generated using construct pΔ_Mtb_*ribC;* unmarked mutant generated using allelic exchange | This study |
| Mtb Δ*ribC*::*ribC* | Complemented mutant generated by introducing construct pMV_Mtb_*ribC* in strain Mtb Δ*ribC;* Hyg^R^ | This study |

**References**

1. Snapper SB, Melton RE, Mustafa S, Kieser T, Jacobs WR, Jr. Isolation and characterization of efficient plasmid transformation mutants of *Mycobacterium smegmatis. Mol Microbiol.* 1990;4(11):1911-9.
2. Ioerger TR, Feng Y, Ganesula K, Chen X, Dobos KM, Fortune S, et al. Variation among genome sequences of H37Rv strains of *Mycobacterium tuberculosis* from multiple laboratories. *J Bacteriol.* 2010;192(14):3645-53.
