# Supplementary material for: Disruption of riboflavin biosynthesis in mycobacteria establishes riboflavin pathway intermediates as key precursors of MAIT cell agonists
Source: PLoS Pathog. 2025 Jul 1;21(7):e1012632. doi: 10.1371/journal.ppat.1012632 (PMC12240317; doi:10.1371/journal.ppat.1012632)
Supplement: S4 Table — (DOCX) [file ppat.1012632.s017.docx]

**S4 Table.** Oligonucleotides used in the study to create gene knockouts and complement strains

| **Primer name** | **Sequence (5’-3’)** | **Application** |
| --- | --- | --- |
| MSMRibA2pTwGFwd | GTGTGAGCTCGTGGAGAAGGGATCGATCACC | Msm *ribA2* complementation |
| MSMRibA2pTwGRev | GTGTGAGCTCTCTCGGGCAGATCGGGGA |  |
| MSMRibGpTwGFwd | GTGTGAGCTCCCTGCTGGTGATGTCGGTC | Msm *ribG* complementation |
| MSMRibGpTwGRev | GTGTGAGCTCGTGTACCGAGAACAGGGCATG |  |
| MSMRibH1pTwGFwd | GTGTGAGCTCCATCGGCGCACAGATCCT | Msm *ribH1* complementation |
| MSMRibH1pTwGRev | GTGTGAGCTCGAAAATCGGTGTGCGGTAAGG |  |
| MSMRibCpMVFwd | GTGTGCGGCCGCCGTCGAGCTTCTCCACGGG | Msm *ribC*  complementation |
| MSMRibCpMVRev | GTGTAAGCTTTTACCGACCCGTACCCGACAT |  |
| MSMRibH2pL5Fwd | gtgttctagagagtggatggtgggcgga | Msm *ribH2* complementation |
| MSMRibH2pL5Rev | gtgtctgcagtatcaccacacaccccgcttc |  |
| MSMRibCKO_UpFwd | GTGTAAGCTTCAGCGGACCGATCACCAG | Msm *ribC* gene knockout |
| MSMRibCKO_UpRev | GTGTGCATGCCAGTACGCCCAGTTCTTCAAC |  |
| MSMRibCKO_DwFwd | GTGTGCATGCGTCATCGCGAAATACGTTGAG |  |
| MSMRibCKO_DwRev | GTGTGAGCTCGCGGCCGCCTCATCCAGCGACACACGGG |  |
| MtbRibA2pTwGFwd | GTGTGAGCTCGTTCGGCCGTCTGATCGA | Mtb *ribA2* complementation |
| MtbRibA2pTwGRev | GTGTGAGCTCCAGATCTTTCCGTGCCAGCTG |  |
| MtbRibCpMVFwd | GTGTGCGGCCGCGTACAGGATCCCGTCGAACAC | Mtb *ribC*  complementation |
| MtbRibCpMVRev | GTGTAAGCTTGATCTCGTCCGGAATGCAAGA |  |
| MtbRibC_KO_UpFwd | GTGAAGCTTGAAACGTTCCCAACCGAAGAA | Mtb *ribC* gene knockout |
| MtbRibC_KO_UpRev | GTGGCTAGCGGCGGTAACCATCGGACC |  |
| MtbRibC_KO_DwFwd | GTGGCTAGCGTAGTCGCAAAGTATGTTGAGC |  |
| MtbRibC_KO_DwRev | GTGGCGGCCGCTGAAATCGTCGGCCACACTG |  |
| MtbFbiC_KO_UpFwd | gtgtaagcttgtgccaacctgaccaccg | Mtb *fbiC* gene knockout^*^ |
| MtbFbiC_KO_UpRev | gtgtactagttacccgccgcaacgctga |  |
| MtbFbiC_KO_DwFwd | gtgtactagtggcacgctgatggaggag |  |
| MtbFbiC_KO_DwRev | gtgtgcggccgcgactttatcggtggacagtttgac |  |
| MSMRibA2_ORB_KO | GACGCGTCGATCTCGGGCAGATCGGGGACGCCGACGCCGCT  CACTGCGCCCCGCCCGATTCCGTCGGCCTGGTTTGTCTGGTC  AACCACCGCGGTCTCAGTGGTGTACGGTACAAACCCGCCCT  CTCGACGGAATCGAGCCTGGTCATAATCGCCACCTTGCCTG  TTTCTTGCCTTTTCGAGGGCGGA | Msm *ribA2* gene knockout |
| MSMRibG_ORB_KO | ATGGGCCCGCGTGAGGCAGTTAACTCGAACGCGGTCGCCC TCAGTTGGGGACCAGGCTCATCAGGACGTCGGTTTGTCTGG  TCAACCACCGCGGTCTCAGTGGTGTACGGTACAAACCCCGC  ATCGCGGCCTCGACCGAGATGCTCACAACGACAGGTGCTTC  GAGGCTCCGGCCGCCTGCCTGCGCA | Msm *ribG* gene knockout |
| MSMRibH_ORB_KO | TGCACGTCCCAGGTCTCGTCCGCCATCTGTGAGGCCATCGT  CACGCGCGGCGCAATTCGCGCAGCGTCACGGTTTGTCTGGT  CAACCACCGCGGTCTCAGTGGTGTACGGTACAAACCGGGCA  GATCGGGGACGCCGACGCCGCTCACTGCGCCCCGCCCGATT  CCGTCGGCCTGCGGTCACCCAGCA | Msm *ribH* gene knockout |
| MtbRibA2_ORB_KO | GCATCCAGCGACGGCAGATCCGGCACCCCGGCGCCACCCTT  CACAAGGCACCGCCGAATTCTCCGGGCAGGGTTTGTCTGGT  CAACCACCGCGGTCTCAGTGGTGTACGGTACAAACCCGCCC  GCTCGACGGAGTCCAACCTCGTCATCTTTGCTACCTAGCTGT  CAGGCCTGGCCGCACGCGAAGCC | Mtb *ribA2* gene knockout |
| MtbRibH_ORB_KO | TGAGGACGCAACACGACGTCCCAGTCGTTCGGTGCGGCGGT  CACGAGTGAGCGCGCAGCTCGCGCAGGGTGGTTTGTCTGGT  CAACCACCGCGGTCTCAGTGGTGTACGGTACAAACCCAGAT  CCGGCACCCCGGCGCCACCCTTCACAAGGCACCGCCGAATT  CTCCGGGCAGATGCACGGATTCGT | Mtb *ribH* gene knockout |
| MtbRibG_ORB_KO | TCCAGGAGCAACCGACCCTGGCGCCCCAAGTGGAGCCGCTCTAACGAGCCACCAAGCTCAGCAACAGATCGGTTTGTCTGGTCAACCACCGCGGTCTCAGTGGTGTACGGTACAAACCGTCGATGCTCTTGACCTGCTCCACGTTCATAGGCTCAGGTGGAGTGAGGCGGCACCGGCCTGTCGCCGTA | Mtb *ribG* gene knockout^*^ |

^*-^ unable to create the gene deletion strains
