# Supplementary material for: Disruption of riboflavin biosynthesis in mycobacteria establishes riboflavin pathway intermediates as key precursors of MAIT cell agonists
Source: PLoS Pathog. 2025 Jul 1;21(7):e1012632. doi: 10.1371/journal.ppat.1012632 (PMC12240317; doi:10.1371/journal.ppat.1012632)
Supplement: S5 Table — (DOCX) [file ppat.1012632.s018.docx]

**S5 Table.** Primers used for qRT-PCR analysis

| **Primer name** | **Sequence (5’-3’)** | **Amplicon size (bp)** | **Gene region amplified** |
| --- | --- | --- | --- |
| qRT_MSM_3072Fwd | GAGGGCGACCTGATCTT | 95 | MSMEG_3072 (*ribA2*) |
| qRT_MSM_3072Rev | TCGAGCGGAACACACA |  |  |
| qRT_MSM_3067Fwd | ATCGATCAGGCCGAACA | 75 | MSMEG_3067 (*ribG*) |
| qRT_MSM_3067Rev | GTCGCGATCGAGGATCA |  |  |
| qRT_MSM_3073Fwd | ATCGAGATCCCGGTCGT | 113 | MSMEG_3073 (*ribH1*) |
| qRT_MSM_3073Rev | ACGGCATCACACACATAGT |  |  |
| qRT_MSM_3071Fwd | GTGAACGGCGTATGTCTC | 85 | MSMEG_3071 (*ribC*) |
| qRT_MSM_3071Rev | ACCGGTTGAGCGTCT |  |  |
| qRT_MSM_2653Fwd | GATCGACGTGTTCCTGGTGAT | 163 | MSMEG_2653 (*ribF*) |
| qRT_MSM_2653Rev | CAGCATGTCGACGTTGCC |  |  |
| qRT_MSM_6598Fwd | ACGTTGGAGTTCTTCGAGGTG | 135 | MSMEG_6598 (*ribH2*) |
| qRT_MSM_6598Rev | GAACTCGTGCCGGTAGATGC |  |  |
| qRT_MSM_5126Fwd | CTGTCCATCCCGTTCACCAC | 131 | MSMEG_5126 (*fbiC*) |
| qRT_MSM_5126Rev | TTCTGCACGATCACTTCCTGG |  |  |
| qRT_MSM_2758Fwd | ACCAAGGGCTACAAGTTCTCG | 198 | MSMEG_2758 (*sigA*) |
| qRT_MSM_2758Rev | CATCTCCTTGGCGAGCTCTTC |  |  |
| qRT_Rv1415Fwd | CACGGAATGGCATTGGAACTG | 144 | Rv1415 (*ribA2*) |
| qRT_Rv1415Rev | GCAGAACCCCACCATCCTTG |  |  |
| qRT_Rv1409Fwd | CTCTGATCGAAGCCAGGGTG | 199 | Rv1409 (*ribG*) |
| qRT_Rv1409Rev | TACTTCCAGGTGACATGCGG |  |  |
| qRT_Rv1416Fwd | AATTGGCCCGCAATCATGATG | 130 | Rv1416 (*ribH*) |
| qRT_Rv1416Rev | GGCGTCGAGGAATCCAGC |  |  |
| qRT_Rv1412Fwd | ACATCGTGCAGGGACATGTG | 123 | Rv1412 (*ribC*) |
| qRT_Rv1412Rev | AGCCCTTTTCGACGACATAGC |  |  |
| qRT_Rv2786Fwd | GCTTGGTTCACGGTGCTC | 103 | Rv2786c (*ribF*) |
| qRT_Rv2786Rev | CGGAGAAGGTGGGATTGGTC |  |  |
| qRT_Rv2703Fwd | CGGTGATTTCGTCTGGGATGA | 113 | Rv2703 (*sigA*) |
| qRT_Rv2703Rev | TGCCGATCTGTTTGAGGTAGG |  |  |
