# Supplementary material for: Disruption of riboflavin biosynthesis in mycobacteria establishes riboflavin pathway intermediates as key precursors of MAIT cell agonists
Source: PLoS Pathog. 2025 Jul 1;21(7):e1012632. doi: 10.1371/journal.ppat.1012632 (PMC12240317; doi:10.1371/journal.ppat.1012632)
Supplement: S6 Table — (DOCX) [file ppat.1012632.s019.docx]

**S6 Table.** Window scheme used for DIA-PASEF acquisition

| #MS Type | Cycle Id | Start IM [1/K0] | End IM [1/K0] | Start Mass [m/z] | End Mass [m/z] | CE [eV] |
| --- | --- | --- | --- | --- | --- | --- |
| MS1 | 0 | - | - | - | - | - |
| PASEF | 1 | 1.3806 | 1.4433 | 1385.82 | 1435.82 | - |
| PASEF | 1 | 1.2112 | 1.38 | 1091.82 | 1141.82 | - |
| PASEF | 1 | 0.9869 | 1.1987 | 748.82 | 798.82 | - |
| PASEF | 1 | 0.795 | 0.9617 | 503.82 | 553.82 | - |
| PASEF | 1 | 0.7101 | 0.7657 | 307.82 | 357.82 | - |
| PASEF | 2 | 1.4088 | 1.4433 | 1434.82 | 1484.82 | - |
| PASEF | 2 | 1.2394 | 1.4039 | 1140.82 | 1190.82 | - |
| PASEF | 2 | 1.0234 | 1.2305 | 797.82 | 847.82 | - |
| PASEF | 2 | 0.8334 | 1.0106 | 552.82 | 602.82 | - |
| PASEF | 2 | 0.7063 | 0.8147 | 356.82 | 406.82 | - |
| PASEF | 3 | 1.2959 | 1.4433 | 1238.82 | 1288.82 | - |
| PASEF | 3 | 1.0949 | 1.2844 | 895.82 | 945.82 | - |
| PASEF | 3 | 0.8718 | 1.0596 | 601.82 | 651.82 | - |
| PASEF | 3 | 0.7183 | 0.8637 | 405.82 | 455.82 | - |
| PASEF | 4 | 1.183 | 1.3561 | 1042.82 | 1092.82 | - |
| PASEF | 4 | 0.9485 | 1.1576 | 699.82 | 749.82 | - |
| PASEF | 4 | 0.7567 | 0.9127 | 454.82 | 504.82 | - |
| PASEF | 5 | 1.3241 | 1.4433 | 1287.82 | 1337.82 | - |
| PASEF | 5 | 1.1266 | 1.3083 | 944.82 | 994.82 | - |
| PASEF | 5 | 0.9101 | 1.1086 | 650.82 | 700.82 | - |
| PASEF | 6 | 1.2677 | 1.4278 | 1189.82 | 1239.82 | - |
| PASEF | 6 | 1.0591 | 1.2604 | 846.82 | 896.82 | - |
| PASEF | 7 | 1.3523 | 1.4433 | 1336.82 | 1386.82 | - |
| PASEF | 7 | 1.1548 | 1.3322 | 993.82 | 1043.82 | - |

*IM; Ion Mobility, CE; Collision Energy
