# Supplementary material for: Disruption of riboflavin biosynthesis in mycobacteria establishes riboflavin pathway intermediates as key precursors of MAIT cell agonists
Source: PLoS Pathog. 2025 Jul 1;21(7):e1012632. doi: 10.1371/journal.ppat.1012632 (PMC12240317; doi:10.1371/journal.ppat.1012632)
Supplement: S7 Table — (DOCX) [file ppat.1012632.s020.docx]

**S7 Table.** Peptides used for targeted proteomics by PRM-MS

| **Protein** | **Abbreviation** | **Peptide (m/z)** | **Transitions** | **Retention time (min)** | **LOD (nM)** | **LOQ (nM)** |
| --- | --- | --- | --- | --- | --- | --- |
| RibC  (Rv1412) | ELT | H-ELTTLGSAAVGTR^-OH  638.3488++ | T [y11] - 1043.5719+  T [y10] - 942.5242+  L [y9] - 841.4766+  G [y8] - 728.3925+  S [y7] - 671.3710+  A [y6] - 584.3390+] | 17.812 | 3.37 | 10.22 |
| RibH  (Rv1416) | GGA | H-GGAGVPDLPSLDASGVR^-OH  784.4074++ | P [y12] - 1236.6458+  L [y10] - 1024.5661+  P [y9] - 911.4820+  D [y6] - 614.3132+  V [y13] - 668.3608++  P [y12] - 618.8265++ | 24.723 | 6.04 | 18.31 |
|  | LAI | R.H-LAIVASSWHGK^-OH.I  584.8273++ | I [y9] - 992.5403+  V [y8] - 879.4563+  A [y7] - 780.3879+  S [y6] - 709.3507+  H [y3] - 349.2074+  A [y10] - 532.2924++ | 16.52 | 13.47 | 40.823 |
