# Supplementary material for: Disruption of riboflavin biosynthesis in mycobacteria establishes riboflavin pathway intermediates as key precursors of MAIT cell agonists
Source: PLoS Pathog. 2025 Jul 1;21(7):e1012632. doi: 10.1371/journal.ppat.1012632 (PMC12240317; doi:10.1371/journal.ppat.1012632)
Supplement: S8 Table — (DOCX) [file ppat.1012632.s021.docx]

**S8 Table.** Peptides used for targeted proteomics by PRM-MS and concentration used in final assay

| **Protein** | **Precursor** | **Peptide Sequence** | **Concentration (pmol/µl)** |
| --- | --- | --- | --- |
| Rv1416\|ribH | 784.4074++ | GGAGVPDLPSLDASGVR | 0.15625 |
| Rv1416\|ribH | 584.8273++ | LAIVASSWHGK | 0.0390625 |
| Rv1412\|ribC | 638.3488++ | ELTTLGSAAVGTR | 0.01953125 |
