# Supplementary material for: Disruption of riboflavin biosynthesis in mycobacteria establishes riboflavin pathway intermediates as key precursors of MAIT cell agonists
Source: PLoS Pathog. 2025 Jul 1;21(7):e1012632. doi: 10.1371/journal.ppat.1012632 (PMC12240317; doi:10.1371/journal.ppat.1012632)
Supplement: S9 Table — (DOCX) [file ppat.1012632.s022.docx]

**S9 Table.** Precursors and transition ions used for targeted metabolomics by MRM-MS

| **Compound** | **Abbreviation** | **Precursor m/z** | **Transitions m/z** | **Retention Time (Min) 100 mm column** | **Retention Time (min) 150 mm column** | **LOD (ng/ml)** | **LOQ (ng/ml)** |
| --- | --- | --- | --- | --- | --- | --- | --- |
| Riboflavin | RF | 377.2 | 243.1 | 5.6 | 6.2 | 1.949 | 5.907 |
|  |  |  | 192 |  |  |  |  |
|  |  |  | 172 |  |  |  |  |
| Dimethyl-ribityl lumazine | DMRL | 327.1 | 193 | 1.9 | 2.5 | 40.3992 | 122.4218 |
|  |  |  | 148 |  |  |  |  |
|  |  |  | 69.2 |  |  |  |  |
| 5-amino ribitylaminouracil | 5-A-RU | 277.1 | 155 | NA | 1.3 | NA | NA |
|  |  |  | 69.2 |  |  |  |  |
|  |  |  | 57.2 |  |  |  |  |
