# Supplementary material for: Disruption of riboflavin biosynthesis in mycobacteria establishes riboflavin pathway intermediates as key precursors of MAIT cell agonists
Source: PLoS Pathog. 2025 Jul 1;21(7):e1012632. doi: 10.1371/journal.ppat.1012632 (PMC12240317; doi:10.1371/journal.ppat.1012632)
Supplement: S10 Table — (DOCX) [file ppat.1012632.s023.docx]

**S10 Table. Confirmation of riboflavin pathway mutants by WGS**

| **Strain** | **% Coverage** | **Mean depth** |
| --- | --- | --- |
| Msm Δ*ribA2::ribA2* | 99.9993 | 66.5371 |
| Msm Δ*ribA2* | 99.9776 | 36.4000 |
| Msm Δ*ribC::ribC* | 99.9992 | 63.2161 |
| Msm Δ*ribC* | 99.9919 | 63.2161 |
| Msm Δ*ribG::ribG* | 99.9989 | 52.7297 |
| Msm Δ*ribG* | 99.9838 | 42.7425 |
| Msm Δ*ribH1::ribH1* | 99.9996 | 67.0717 |
| Msm Δ*ribH1* | 99.9941 | 52.7297 |
| Msm Δ*fbiC::fbiC* | 99.9800 | 24.1001 |
| Msm *ΔfbiC* | 99.9560 | 32.0029 |
| Msm Δ*ribH2*::*ribH2* | 99.9842 | 28.5218 |
| Msm Δ*ribH2* | 99.9817 | 25.3715 |

| **Strain** | **% Coverage** | **Mean depth** |
| --- | --- | --- |
| Mtb Δ*ribA2::ribA2* | 99.9084 | 94.0475 |
| Mtb Δ*ribA2* | 99.9180 | 82.0263 |
| Mtb Δ*ribC::ribC* | 99.9574 | 116.268 |
| Mtb Δ*ribC* | 99.9475 | 66.6217 |
| Mtb Δ*ribH::ribH* | 99.9453 | 93.5616 |
| Mtb Δ*ribH* | 99.9292 | 82.5667 |
