# Supplementary material for: Disruption of riboflavin biosynthesis in mycobacteria establishes riboflavin pathway intermediates as key precursors of MAIT cell agonists
Source: PLoS Pathog. 2025 Jul 1;21(7):e1012632. doi: 10.1371/journal.ppat.1012632 (PMC12240317; doi:10.1371/journal.ppat.1012632)
Supplement: S11 Table — (DOCX) [file ppat.1012632.s024.docx]

**S11 Table. SNPs detected in Msm knockout mutants and not in wild type***

| **Reference**** | **Position** | **Strain** | **Base Change** | **Gene** | **Full Name** | **SNPs** | **AA Change** | **Syn/Non-Syn** |
| --- | --- | --- | --- | --- | --- | --- | --- | --- |
| CP000480.1 | 3021459 | Msm Δ*ribA2*  and  Msm Δ*ribC::ribC*  and  Msm Δ*ribC* | G to A | *gabT* | 4-aminobutyrate transaminase | GTG/CAC to ATG/TAC | H to Y | NS |
| CP000480.1 | 5551098 | Msm Δ*ribA2*  and  Msm Δ*ribC*  and  Msm Δ*ribC::ribC* | A to T | MSMEG_5468 | conserved hypothetical protein | GAG/CTC to GTG/CAC | L to H | NS |
| CP000480.1 | 5653976 | Msm Δ*ribH1::ribH1* | T to C | MSMEG_5566 | transcriptional regulator | TAC/ATG to CAC/GTG | V to V | S |
| NZ_CP054795.1 | 6047838 | Msm Δ*fbiC*  and  Msm Δ*fbiC*::*fbiC* | C to T | FOB87_29075 | tripartite tricarboxylate transporter permease | CTG to TTG | L to L | S |
| NZ_CP054795.1 | 2248947 | Msm Δ*ribH1*Δ*ribH2*  and  Msm Δ*ribH1*Δ*ribH2*::  *ribH1 ribH2* | C to A | FOB87_10845 | sigma-70 family RNA polymerase sigma factor | CAG to AAG | Q to K | NS |
| NZ_CP054795.1 | 719556 | Msm Δ*ribH1*Δ*ribH2*::  *ribH1 ribH2* | A to G | IR | Intergenic Region |  |  |  |
| NZ_CP054795.1 | 4536517 | Msm Δ*ribH2*  and  Msm Δ*ribH2*::*ribH2* | G to T | *pcrA* | DNA helicase PcrA | GCC/CGG to TCC/AGG | G to G | S |

* Only showing SNPs unique to each analyzed strain in comparison to wild type. SNPs shared between mutant and wild type strains when compared to mc^2^155 reference genome are not shown.

** Analysis of strains initially compared against *Mycobacterium smegmatis* reference genome (CP000480.1) and strains created later were analyzed against the updated reference genome of *Mycolicibacterium smegmatis* (NZ_CP054795.1)
