# Supplementary material for: Disruption of riboflavin biosynthesis in mycobacteria establishes riboflavin pathway intermediates as key precursors of MAIT cell agonists
Source: PLoS Pathog. 2025 Jul 1;21(7):e1012632. doi: 10.1371/journal.ppat.1012632 (PMC12240317; doi:10.1371/journal.ppat.1012632)
Supplement: S12 Table — (DOCX) [file ppat.1012632.s025.docx]

**S12 Table. SNPs detected in Mtb mutants and not in wild type**

| **Reference** | **POS** | **Strain** | **Base Change** | **Gene** | **Full Name** | **SNPs** | **AA Change** | **Syn/Non-Syn** |
| --- | --- | --- | --- | --- | --- | --- | --- | --- |
| NC_000962.3 | 3247686 | Mtb Δ*ribA2*  and  Mtb Δ*ribA2*::*ribA2*  and  Mtb Δ*ribH*  and  Mtb Δ*ribH*::*ribH* | C to T | *ppsA* | phthiocerol synthesis polyketide synthase type I PpsA | CTG to TTG | L to L | S |
| NC_000962.3 | 3247697 | Mtb Δ*ribA2*  and  Mtb Δ*ribA2*::*ribA2*  and  Mtb Δ*ribH*  and  Mtb Δ*ribH*::*ribH* | G to A | *ppsA* | phthiocerol synthesis polyketide synthase type I PpsA | CCG to CCA | P to P | S |
| NC_000962.3 | 3247694 | Mtb Δ*ribA2*  and  Mtb Δ*ribA2*::*ribA2* | A to G | *ppsA* | phthiocerol synthesis polyketide synthase type I PpsA | GCA to GCG | A to A | S |
| NC_000962.3 | 3247901 | Mtb Δ*ribA2*  and  Mtb Δ*ribA2*::*ribA2* | G to C | *ppsA* | phthiocerol synthesis polyketide synthase type I PpsA | GTG to GTC | V to V | S |
| NC_000962.3 | 3247928 | Mtb Δ*ribA2*  and  Mtb Δ*ribA2*::*ribA2* | T to G | *ppsA* | phthiocerol synthesis polyketide synthase type I PpsA | GCT to GCG | A to A | S |
| NC_000962.3 | 3247929 | Mtb Δ*ribA2*  and  Mtb Δ*ribA2*::*ribA2* | T to C | *ppsA* | phthiocerol synthesis polyketide synthase type I PpsA | TTG to CTG | L to L | S |

* Only showing SNPs unique to each analyzed strain in comparison to wild type. SNPs shared between mutant and wild type strains when compared to H37Rv reference genome are not shown.
