# Supplementary material for: Disruption of riboflavin biosynthesis in mycobacteria establishes riboflavin pathway intermediates as key precursors of MAIT cell agonists
Source: PLoS Pathog. 2025 Jul 1;21(7):e1012632. doi: 10.1371/journal.ppat.1012632 (PMC12240317; doi:10.1371/journal.ppat.1012632)
Supplement: Attachment — Submitted filename: Response_to_reviewers_FINAL_PPATHOGENS-D-24-02117R1.pdf [file ppat.1012632.s031.pdf]

RESPONSE TO REVIEWERS' COMMENTS - PPATHOGENS-D-24-02117

Reviewer #1:

Reviewer #1: Chengalroyen and Oketade et al generate knock-out mutants of genes involved in riboflavin biosynthesis in Mycobacterium smegmatis and M. tuberculosis and reconstitute knock-outs with the relevant genes. They evaluate the impact of the mutants on the growth of the bacteria in the presence and absence of riboflavin, quantify the impact of individual knock-outs on riboflavin biosynthesis gene transcripts and protein levels and test their capacity to stimulate previously generated M. smegmatis and M. tuberculosis-specific MR1-reactive T cell clones. They confirm a central role for 5-A-RU in M. smegmatis and M. tuberculosis-derived MAIT cell antigens. They also argue that residual activation by mutants of RibA2 or RibG, required for the generation of 5-A-RU, suggests an alternate source/s of minor mycobacterial MAIT cell agonists. They found that deletion of RibH which catalyses the conversion of 5-A-RU and 3,4-DHBP to DMRL enhanced MAIT cell recognition of M. tuberculosis but had no impact on the MAIT cell response to M. smegmatis.
Altogether this study adds to similar studies pinpointing 5-A-RU as the key intermediate in riboflavin biosynthesis antigen generation in a range of bacteria. Mycobacteria is of clinical relevance and the study is hence important. My main concerns relate to some of the mutants generated where reconstitution was little or not successful, shedding doubt on the specificity of the mutants and hence their interpretations of T cell activation. For the T cell activation experiments raw activation data needs to be shown, stats to wild-type bacteria activation done and MR1 dependency tested. These experiments were sometimes over-interpreted, especially for the TRAV1-2 neg T cell clone where the error bars are too large for some mutants to interpret the data at all.
Response: We thank the reviewer for the positive comments, and for highlighting key issues, which are addressed below.

1) For experiments in Fig. 2, Fig. 3 only 2 biological replicates are shown and SD is plotted. SD can only be plotted for 3 or more biological replicates and experiments need to be done at least 3 times. How come Fig S3 says it shows data from 3 biological replicates, but more than 3 dots are seen in each bar and why do the number of dots differ between bars in the same graphs? Also, in Fig S4 it says data is from 3 independent experiments but in the bar chart there are 4 dots in each bar. In Fig. 3, the Figure legend states that there were 3 biological replicates for A, C and E but for the DIA measurements the number of biological replicates was not indicated (although here the number of dots in the bars suggests it has been done 3-times).
   Response: We repeated all proteomics experiments to include 3 biological replicates and two technical replicates. For figures in supplementary, we ensure all individual dots (representing each replicate) is visible. We clarified number of samples (biological or technical) for these experiments in the methods section and legend. For the growth kinetics experiments we also repeated to include 4 biological replicates.

2) In all cases not only mean values but also values from individual experiments should be shown. This was done for most Figures but please amend this also for Fig. 3 and 4 A, C, E.
   Response: We show the mean values for Fig 3 - 5 based on the analyses conducted for qRT-PCR. We followed the workflow described at the following link for the fold change analysis:

https://www.science.smith.edu/cmbs/wp-content/uploads/sites/36/2015/09/Analyzing-your-QRT-for-relative-2%5E-%E2%88%86%E2%88%86Ct.pdf.
We now include the raw data used for the generation of the means and subsequent figures in the file, S1 Data.

3) Fig. 2 requires stats.
Response: Corrected. Statistical comparisons, against wildtype were performed using a one-way ANOVA and Dunnett's multiple comparison test.

4) In Fig. 3, 4, S3 it looks like stats were only done between mutant+RF versus reconstituted mutant but should also be done between mutant+RF and Msm WT+RF.
Response: Fig 3-5 are corrected to include these statistical analyses. S4 Fig is a quantification of our observations in Figs 3-4. Data used to generate all these figures is now provided in S1 and S2 Data files.

5) Fig 4 B, D, F –it looks like the data for the condition Mtb WT+RF was omitted from the graph whilst it appears in the legend. Accordingly, I cannot comment on text in lines 210 and 212 wrt protein levels.
Response: We now include data for Mtb WT with riboflavin supplement in Fig 5.

6) Fig. 5, 6 The absolute IFNg production of each T cell clone to wt Msm and in response to a pan-stimulus like PMA/Ionomycin or anti-CD3/CD28 also needs to be shown.
Response: We now include the IFNγ spot forming units of each T cell clone to WT Msm and PHA in S12 Fig.

7) Why is there only limited to no complementation observed for the Msm ΔribA2, ΔribH1 and ΔribH2ΔribH1 strains based on growth kinetics (Fig. 2) and transcript and protein of the relevant genes (Fig. 3) as well as T cell activation (Fig. 5 for ribH2, alone or in combination with ribH1)? Was the reconstitution done several times? The reconstitution is an important verification of the mutant and when complementation does not work this casts doubt on the specificity of the mutants.
Response: As we reported previously (https://pubmed.ncbi.nlm.nih.gov/38916330/), in Msm, the riboflavin biosynthesis genes lie on an operon comprising three genes: *ribC-ribA2-ribH*. In constructing the complementation vectors, we amplified the corresponding gene's ORF together with ~300 bp of upstream (5') sequence to include the putative promoter region nested within the operon. In all three complemented strains, Msm Δ*ribA2*::*ribA2*, Msm Δ*ribH1*::*ribH1* and Δ*ribH2*Δ*ribH1*::*ribH2 ribH1,* our first test of whether the complementing genes introduced into the knockout mutant backgrounds were expressed was whether the complemented derivatives were able to grow on solid media in the absence of exogenous riboflavin supplement. While this was confirmed in all three cases, the growth of these strains on solid media was noticeably retarded relative to the parental wildtype strain. Subsequent comparative assessment of the growth kinetics of these strains in liquid media confirmed that these strains were indeed attenuated for growth relative to the wildtype. We state in the manuscript, Ln 180-182: "Additionally, we attributed this effect to comparatively weak expression of the complementing genes, *ribA2* and *ribH1* from nested promoters instead of the main operonic promoter (located upstream of *ribC*)". This conclusion was confirmed by qRT-qPCR analysis which revealed that the transcript levels of the complementing *ribA2* and *ribH1* genes

introduced back into the mutant strains were lower than their wildtype levels. The text has been revised to explain this point, Ln 222-231: "Msm Δ*ribH2* Δ*ribH1*::*ribH2 ribH1* showed retarded growth kinetics, which improved to some extent in the presence of riboflavin albeit not to the level of the wildtype. qRT-PCR analysis confirmed that the level of *ribH1* transcript was lower in the Msm Δ*ribH2*Δ*ribH1*::*ribH2 ribH1* strain than in wildtype (**Fig 4A**), which could potentially explain the growth impairment of this complemented strain **(Fig 2F)**. Whole genome sequencing (WGS) revealed only two non-synonymous SNPs in Msm Δ*ribH2* Δ*ribH1*::*ribH2 ribH1* which were situated in a σ^{70} family RNA polymerase sigma factor and an intergenic region, respectively. The contributions thereof (if any) to the growth phenotype of the complemented double mutant and incomplete growth restoration by exogenous riboflavin, are unclear." Furthermore, in Ln 351-3503: "Deletion of *ribH1* and *ribH2* alone or in combination had no impact on recognition of Msm by the three TRAV1-2+ clones, D426-G11, D481-C7 and D481-F12."

8) Fig. S2 assesses growth phenotyping upon streaking. It is difficult to interpret the plate images given that too many bacteria have been streaked out and individual colonies cannot be identified. The only conclusion that can be drawn from the data presented is of qualitative nature - bacteria grew or not. Instead, the authors should quantify the numbers of colonies and the size of the colonies upon plating a certain volume of bacteria (rather than streaking them out) to determine the growth phenotype. Letting the bacteria grow for a shorter time frame would allow for individual colonies to be identified the size of which can be measured.
   Response: This experiment has been done as is shown in S2 and S3 Fig. Text describing these data for Msm has been added in Ln 175-180: "When plating the wildtype and complemented Msm auxotrophs on 7H10 agar with or without riboflavin supplementation, there was no significant difference in total CFU counts (**S2 Fig B**). However, colonies of the complemented derivatives Msm Δ*ribA2*::*ribA2*, Δ*ribH1*::*ribH1*, Δ*ribH2*Δ*ribH1*::*ribH2* and Δ*ribH2*Δ*ribH1*::*ribH2 ribH1* emerged later than the other strains (**S2 Fig A**) and were significantly smaller than wildtype (**S2 Fig C**)." And, for the Mtb data, the following text is included, Ln 192-195: "Likewise, CFU counts of the knockout mutants grown on riboflavin, and of the complemented strains were equivalent to wildtype (**S3 Fig B**) although the colony size of the wildtype strain was on average smaller than the other strains (**S3 Fig C**)."

9) Shouldn't also the ΔribH2 mutant of Msm be listed amongst those mutants that showed no growth phenotype and the ΔribH1 mutant of Msm amongst those mutants that were auxotrophic for riboflavin?
   Response: The Msm Δ*ribH2* and Δ*ribH1* single gene deletion mutants are not riboflavin auxotrophs; this point has been clarified in Ln 186-192: "The growth of Msm Δ*ribH1* observed on solid media (**S2 Fig A)** and in liquid culture – albeit more slowly than the wildtype (**Fig 2D**) – confirmed the viability of this mutant in the absence of riboflavin and hence, an alternate source/s of lumazine synthase activity in Msm that can compensate, albeit partially, for the loss of RibH1. The fact that deletion of both *ribH1* and *ribH2* rendered Msm incapable of growth on solid media or in liquid culture in the absence of riboflavin supplement is consistent with the designation of RibH2 as an alternate lumazine synthase in Msm."

10) Why were no mutant KO's of ribG and fbiC for Mtb generated?

Response: Repeated attempts to create a Mtb Δ*ribG* knockout mutant using the ORBIT method were unfortunately unsuccessful. PCR screening of ~50 colonies isolated from these experiments failed to identify any clones with the expected deletion mutant genotype. We hypothesized that the oligo preferentially bound to another region with high sequence similarity in the genome. Likewise, repeated attempts to create a Mtb Δ*fbiC* knockout mutant by allelic exchange mutagenesis using a suicide plasmid harbouring homologous regions ~1674 bp upstream and 1648 bp downstream in length flanking the deletion leaving 78 amino acid of the FbiC ORF were unsuccessful. In this case, no single crossover homologous recombinants were identified from 4 independent electroporations of the suicide plasmid into Mtb. Allelic exchange and ORBIT were not, however, used as alternate mutagenesis approaches for constructing Δ*ribG* and Δ*fbiC* mutants of Mtb, respectively. Based on this information, we respectfully submit that doing so would fall beyond the scope of the study. The following text was therefore included, Ln 532-533: "In contrast, for reasons that are unclear, attempts to generate a knockout mutant of *fbiC* in Mtb were unsuccessful" and in Ln 541-544: "The ORBIT system [34] was used to create individual gene knockouts of *ribA2*, *ribG* or *ribH* in Msm and *ribA2* or *ribH* in Mtb although an attempt to construct a *ribG* knockout in Mtb using ORBIT was unsuccessful." The oligos used to try to create these knockouts strains were added to S4 Table.

11) The hypothesised redundancy of the lumazine synthase activity in Msm should be described consistently and in a more cautious way in lines 142 and 148. Fig 2A shows that in Msm deletion of ribH1 but not of ribH2 showed an impact on growth. In addition Fig 3 shows that albeit an increase in transcript and protein level of ribH2 in the ribH1 mutant growth was still impaired. There was only 30% of growth relative to wt in the presence of RF so this indicates little compensation and not as the authors state, 'cannot fully compensate' (line 148). It would hence be better to tone down the statement about the redundancy of ribH1 and ribH2 in line 142 and make this a point in the discussion.

Response: This observation was made in our previous study (Chengalroyen et al., 2024). We have provided a clearer explanation of this result in the text, Ln 178-192: "However, colonies of the complemented derivatives Msm Δ*ribA2*::*ribA2*, Δ*ribH1*::*ribH1*, Δ*ribH2*Δ*ribH1*::*ribH2* and Δ*ribH2*Δ*ribH1*::*ribH2 ribH1* emerged later than the other strains (**S2 Fig A**) and were significantly smaller than wildtype (**S2 Fig C**). The retarded growth of these strains on solid media would explain the limited complementation of growth of these complemented strains in liquid culture in contrast to other complemented derivatives for which restoration of growth approximating the wildtype was observed (**Fig 2A-G**). Additionally, we attributed this effect to comparatively weak expression of the complementing genes, *ribA2* and *ribH1* from nested promoters instead of the main operonic promoter (located upstream of *ribC*). The growth of Msm Δ*ribH1* observed on solid media (**S2 Fig A)** and in liquid culture – albeit more slowly than the wildtype (**Fig 2D**) – confirmed the viability of this mutant in the absence of riboflavin and hence, an alternate source/s of lumazine synthase activity in Msm that can compensate, albeit partially, for the loss of RibH1. The fact that deletion of both *ribH1* and *ribH2* rendered Msm incapable of growth on solid media or in liquid culture in the absence of riboflavin supplement is consistent with the designation of RibH2 as an alternate lumazine synthase in Msm."

12) In line 238 onwards, how do the authors explain the reduced activation by the reconstituted ΔribH2 mutant of the TRAV1-2- clone, D520-E10 which showed no growth defect in Fig 2 and given the redundancy between ribH1 and ribH2?
Response: We appreciate this comment and find it a valid concern. We have since repeated this a number of times and find the response from the D520-E10 clone variable with regard to the Msm Δ*ribH2* mutant. As a result, we have decided to exclude this clone from the study.

13) 182: 'a 3-fold increase in ribH2 transcript and RibH2 protein was observed in the Msm ΔribH1 mutant suggesting a regulatory interdependence of expression between the two lumazine synthases (Fig 3G and H).' I can see a 3-fold increase in ribH2 transcript but not in protein level in Fig. 3H.
Response. Done. As a result of the colouring of the bar graphs, there might have been a misinterpretation of the data in the original figure. Subsequently, the figure has been reformatted as Fig 4. The text has been revised as follows, Ln 216-220: "Certain knockouts also affected the expression of other members of the pathway, e.g., a < 3-fold increase in *ribH2* transcript and an increase in RibH2 protein was observed in the Msm Δ*ribH1* mutant suggesting a regulatory interdependence of expression between the two lumazine synthases **(Fig 4C and 4D)."**

14) 187 compares the effects of gene knockouts (this study) versus knockdowns (previous study) and this needs to be described and interpreted more accurately: A knock-out mutant of ribF was not analyzed here so cannot be compared. Silencing of ribA2 suppressed the expression of ribG and a trend can also observed in the knock-out. For ribC there is probably no difference. How do the authors explain that silencing of ribA2 suppressed the expression of ribH1 in Msm but there is a significant increase in ribH1 for the knock-out?
Response: (1) *ribF* knockout mutants of Mtb and Msm could not be generated due to the inability to rescue these mutants with the products of RibF (FMN and FAD, as reported previously) in a manner analogous to the chemical complementation of the other riboflavin biosynthesis pathway genes with riboflavin. (2) In our prior work, we showed that in the Msm_*ribA2* hypomorph, CRISPRi-mediated transcriptional repression of *ribA2* has a polar effect consistent with the operonic arrangement (*ribC-ribA2-ribH1*), simultaneously knocking down the expression of *ribH1*. We agree that the upregulation of *ribH1* observed in the Δ*ribA2* mutant is a surprising result. This could be a regulatory mechanism in which the absence of RibA2 would prevent the substrate of RibH, 3,4-DHBP from being generated. An inability to 'sense' the substrate may cause the upregulation of *ribH1* to counter this.

15) 203: 'Additionally, stable isotope-labeled standards were made for peptides derived from RibA2, RibC and RibH' – Fig S3 only has data for RibC and RibH but why not for RibA2?
Response: Targeted PRM analyses included peptides from RibC, RibH and RibA2. However, during our final analysis, the peptide from RibA2 was undetected possibly due to breakdown hence we removed RibA2 from the PRM analysis. Figure and figure legend has been updated to reflect this. Raw data used to generate these figures is available as supplementary S1/S2 Data files.

16) For the TRAV1-2 neg clone D520 E10 the data in Fig. 5 panel D is convincing. However, in panel H activation with the ribH2 from 3 biological replicates ranges from 100% response to 20% of wt+RF, meaning that as it stands it is unclear whether the ko mutant of ribH2 has an impact or not. In the same panel there is a non-significant reduction in the reconstituted fbiC mutant from 90% to less than 25 % which is unexpected but again there is a large range in the data from the 3 experiments. The response by this clone alongside one of the other TRAV1-2+ clones needs to be repeated several times for the ribH2, ribH1+ribH2 and fbiC mutants to really understand the response pattern.
Response: Due to the variability with the response from this clone, we have decided to remove these data from this submission.

17) 319: 'However, residual MAIT cell activation was observed for Msm ΔribA2, Msm ΔribG and Mtb ΔribA2 thus implicating an alternate source/s of minor mycobacterial MAIT cell agonists distinct from the riboflavin pathway.' The mean and range should be quantified and statistics done relative to untreated and anti-MR1 blocked condition. Background activation is the attractive alternative hypothesis and should be described.
Response: To address this concern, we have now used an anti-MR1 antibody to demonstrate that the residual activity in the *ribA2* and *ribG* gene deletion strains is MR1-dependent, shown in S11 Fig. These data would suggest that not all ligands are 5-A-RU dependent, and we have added more granularity to the discussion to reflect this. We can say confidently that these T cell clones are MR1 restricted in that they bind the MR1/5-OP-RU tetramer and do not respond to MR1 knockout cell lines pulsed with Msm or Mtb, and respond to both canonical and non-canonical MR1 antigens (Gold et. Al, *PLOS Biology* (2010), Harriff et al, *Sci Immunol* (2018); Narayanan et al, *Sci. Rep* (2020), Culicke et al, *Commun. Biol*. (2024)). Hence, we have not observed bystander activation in these clones.

18) 249: 'The ΔfbiC mutation had no effect on Msm viability (Fig 2) or recognition by the TRAV1-2+ T cell clones, D426-G11 and D481-C7 (Fig 5). However, there was a trend towards reduced recognition of Msm by the TRAV1-2+ clone, D481-F12, and the TRAV19-1 clone, D510-E10 (Fig 5).' I can only see a trend for a reduced response for the reconstituted Msm ΔfbiC mutant in panels G and H (which is unexpected), whilst the means for the mutant itself sit at 100% and 80%.
Response: Thank you for pointing this out. This experiment was repeated, and the data are shown in Fig 7. The Msm Δ*fbiC* deletion mutant had no effect on recognition by the TRAV1-2+ T cell clones, D426-G11, D481-F12 and D481-C7. In addition, as outlined above, the D520-E10 clone has been removed from the manuscript.

19) Accordingly, 320: 'That Msm also preferentially produces lumazine antigens is suggested by the observation that the ablation of FbiC impacted the recognition of Msm by the non-TRAV1-2 T cell clone D520 E10, which recognizes Msm and not Mtb.' Needs to be changed/removed.
Response: We have removed this sentence from the manuscript.

20) For the D520 E10 clone the authors should also test a double mutant for ribA2 or ribG and ribH1 to determine if the 50% reduced activity observed in each case leads to 0% activation when combined. Is the residual 50% response observed for the ribA2, ribC and ribH1 mutants MR1-depdendent? Additional TRAV1-2 neg T cell clones should be tested to see if the pattern of the D520 E10 clone is generalisable for

the response to M. smegmatis.
Response: This clone has been removed from the manuscript.

21) I don't understand the normalisation done. It is essential to also show the raw activation data, as it is possible that the response of the conventional CD8+ D454 H1-2 clone changes for the different mutants. It would be important to test to which extent there is MR1-dependent activation observed to Mtb ΔribH.
Response: These experiments have been repeated with the addition of anti-MR1 antibody blockade, shown in S11 Fig. The raw activation data are shown in S4 Data file. The response to the CD8+ classically restricted T cell clone (D454 H1-2) does change for the different mutants and we have modified the language to reflect this.

22) Fig. S1: Shouldn't ribH2 be indicated here for Msm?
Response: Corrected – the genomic context of *ribH2* was added to S1 Fig.

23) Fig. 6 Figure legend states the use of DCs as APCs whilst the main text mentions the BEAS-2B bronchial epithelial cell line
Response: This has been updated as DC were used.

24) The display of Fig 3, 4, 5, 6 and S3 needs to be improved for clarity: same colour coding for transcript and protein levels in Fig 3, 4. Having x-axis labels instead of a legend would make it easier to digest the data in Fig 3, 4, 5, 6, S3.
Response: We agree and redesigned all the afore-mentioned figures for clarity. In addition, we modified the original Fig 3 into two figures; Fig 3 and Fig 4 to improve the ease of interpretation Fig 3 shows perturbations for *ribA2*, *ribC* and *ribG* and Fig 4 shows the data for the two lumazine synthases in Msm.

25) 45/46 and throughout 5-A-RU vs 5-AR-U, 5-A-RU is usually used
Response: Done – corrected in Ln 50, 356 and throughout.

26) 67 onwards inaccurate referencing: 'MR1-restricted T cells (MR1Ts) are characterized by their dependence on the highly conserved molecule, MR1 [1].' Reference 1 is not adequate for MR1 T cells. MR1 restriction was demonstrated by Treiner et al Nature 2003. Kjer-Nielsen et al Nature 2012 discovered that MR1 presents Vitamin B metabolites but 5-OP-RU as MAIT cell antigen was described in Corbett et al Nature 2014.
Response: This has been updated.

27) 88: references for Salmonella Typhimurium are 7 and 11; for E. coli there is also Soudais et al JI 2015; lastly Lopez-Rodriguez et al JEM 2023 generated a Klebsiella ribD mutant
Response: Done - Ln 90-95: References were added accordingly, and the text revised as follows: "The central role of the microbial riboflavin pathway in MAIT cell activation has been confirmed in different organisms. These include *Lactococcus lactis* (7), *Salmonella Typhimurium* (7, 11), *Escherichia coli* (12, 13), *Streptococcus pneumoniae* (14), *Klebsiella pneumoniae* (15) and *Aspergillus fumigatus* (16) rendered auxotrophic for riboflavin as well as natural variants of *S. Typhimurium* (17) and *S. pneumoniae* (14) with altered expression of the riboflavin pathway."

28) 110: 'in the field of MR-1 (suggesting inserting dependent) immune responses towards Mtb.'
Response: Done – Ln 119-120.

29) 123: 'myriad (insert: of) enzymes'
Response: Done – Ln 137

30) Fig 1B is not really touched on in the main text and not properly described in the Figure legend. It would be of interest to describe the operon organisation and regulation, point out what is not known where applicable and also talk about how the operon organisation and regulation is different to other bacteria.
Response: Done – the figure legend has been adjusted as follows: "Genomic context of the riboflavin biosynthesis genes in Msm and Mtb. Genes coloured in grey are operonic while genes with a diagonal line fill are dispersed in the genome." The text has been revised, Ln 128-132: "In Msm and Mtb the *ribC*, *ribA2* and *ribH* genes are operonic [20], similar to the genomic organisation in *B. subtilis* where the riboflavin biosynthesis genes are arranged on a five-gene operon [21]. In *B. subtilis*, FMN riboswitches regulate the expression of riboflavin biosynthesis and transport [22]; however these regulatory elements have not been identified in the genomes of mycobacteria."

31) 145: 'In prior work, we used a panel of…' check sentence
Response: Done – Ln 159-162.

32) 194: 'Quantification of riboflavin pathway transcript (insert: 'and') protein levels in Msm strains.'
Response: Done – Ln 239

33) 195: explain sigA
Response: Done –The text has been revised, as follows, Ln 584-587: "Transcript levels of the target genes were normalized to the housekeeping gene *sigA* (an essential housekeeping gene which is stably expressed) and calibrated to wildtype Mtb and Msm to calculate the ΔΔCt and determine fold difference in gene expression."

34) 201: cite Fig. 2
Response: Done – Ln 174

35) 206: 'Riboflavin supplement suppressed expression of ribC and enhanced expression of ribA2 in wildtype Mtb (Fig 4A and E) but had no significant impact on expression of other pathway genes (instead: 'had no impact on expression of ribH' as only ribH was tested other than ribA2 and ribC).'
Response: Done – Ln 275

Reviewer #2:

Reviewer #2: The authors of the study conducted experiments on mutants of Mycobacterium smegmatis (Msm) and Mycobacterium tuberculosis (Mtb) to explore the role of genes related to the riboflavin biosynthesis pathway and synthesis of MR1 ligands associated with the activation of MAIT cells. Their findings indicate that when either Msm or Mtb lacks key

enzymes involved in the early or intermediate stages of this pathway, there is a significant reduction in MAIT cell activation. The use of radiolabeled peptides from the RibA2, RibC, and RibH genes were a rigorous and complementary analysis to supplement the gene expression data. The authors show that deletion of RibA2 or RibG resulted in 82-99% reduction in MR1T cell activation, but RibC and RibH deletions did not. Yet RibH deletions did reduce activation of a TRAV1-2- clone, which uniquely recognized Msm (not Mtb), implying it was lumazine-specific. Additionally, the study highlights that the impact on MAIT cell activation differs based on the specific mycobacterial strain involved, particularly when there is a deficiency in the RibH enzyme. Overall, the research underscores a more diverse set of ligands for MR1T cells, and the importance of riboflavin-synthesis intermediates as MR1 ligands for mycobacterial-specific MAIT cell responses. There were only minor issues noted with referencing one of the MR1T clones in the text and in Figure 5. The paper is well-written, clear and straightforward. The model used is well-explained and the use of complemented mutant strains is relevant to their claim.

Response: We thank the reviewer for the positive comments

1) In line #240 the authors describe D510-E10 as a TRAV1-2- clone, but in line #252 they describe it as a TRAV19-1 clone. In line #332, it is listed as non-TRAV1-2 clone 520-E10. In Figure 5, it is also listed as 520-E10. Are these in fact the same MR1T cell clones? If so, it would be helpful to be consistent about how these are named (either with reference to both Va alleles (TRAV1-2- and TRAV19+) or just reference one for clarity.
   Response: These were all references to the same clone. We have since repeated this experiment a number of times and find the response from the D520-E10 clone variable. As a result, we have decided to exclude this clone from the study, as described above in response to comments from Reviewer #1, comment 12.

2) In lines 251-252, the authors state “there was a trend towards reduced recognition of Msm by the TRAC1-2+ clone D481-F12, and the TRAV19-1 clone, D510-E10”. How do the authors explain this effect after fibC deletion in Msm since an antagonist is eliminated and by mass effect, substrate (5-A-RU) should be increased? Is it in fact increased in the fibC mutant? It would enhance this already rigorous dataset and manuscript if the authors could provide these details.
   Response: This sentence has been removed.

3) Line 41: Name of the enzyme for RibH should be listed here as this is the first use of the abbreviation, instead of in the following line 42
   Response: Done – Ln 44

4) Fig.3 Figure color-coded legend is confusing as one giant list. It could be moved to the right of the graphs, or upper right corner for each section to improve reading.
   Response: We agree and have redesigned the figures for clarity.

5) Line #60 authors just mentioned enzyme synthase but did not provide an acronym for LS from Mtb. (Do not consider this super relevant)
   Response: Done – Ln 62-64: The text has been revised as follows: “However, blocking the penultimate step in the pathway, catalyzed by the lumazine synthase, RibH, led specifically to increased MAIT cell recognition of Mtb.”

6) In line 287, RibA2 is listed as both "early" and "penultimate" steps in the riboflavin pathway. Could the authors clarify?
Response: Done – Ln 397-400: The text has been revised as follows: "The dual role of RibA2 is attributable to its bifunctionality in catalyzing both the first step in the pathway via its GTP cyclohydrolase II domain, and, via its 3,4-DHBP synthase domain, the formation of a substrate of RibH, which catalyzes the penultimate step in the pathway."

7) Line #268-269 "[…] normalized these responses to the HLA-B45, Mtb8.4-specific clone." -> Line #278-279 explains the normalization of results by the Mtb8.4-specific CD8 T cell clone. This information could append the sentence initiated in #268 for better understanding.
Response: Done –the text has been revised as follows, Ln 374-377: "As the strains varied in their ability to activate the MAIT cell clones, we normalized these responses to the HLA-B45, Mtb8.4-specific clone, a classically restricted CD8 T cell clone that recognizes Mtb8.4."

8) #320-321 "[…] implicating an alternative source/s of minor mycobacterial MAIT cell agonists […]". Are there any known alternative sources? Supplementary information on the topic would enrich the discussion.
Response: We concur that this is an important topic but are not aware of pathways or ligands that would help us here. This is a really challenging topic, as the current methods for solving non-peptidic ligands are evolving, such that we have largely had to rely on the synthesis and testing of putative antigens.

9) #232 typo; "D481 C7" -> D481-C7
Response: Done – Ln 341

10) #332 typo; "D525 E10" -> D525-E10
Response: This clone has been removed.

Reviewer #3:

Reviewer #3: This a study comparing Mycobacterium smegmatis (Msm) M. tuberculosis (Mtb) viability, growth kinetics, gene expression, and immunogenicity after targeted gene deletion of enzymes in the riboflavin biosynthetic pathway. The manuscript is well-written. The topic is significant as new drug targets are needed against Mtb. Moreover, the riboflavin biosynthetic pathway generates immune ligands for MAIT cells restricted by a highly conserved antigen presenting system in mammals, MR1, and holds potential for immune-directed therapeutics. The authors confirm prior findings, including from the authors themselves, that ribA2, ribG, ribH, and ribC are essential genes in Mtb and further validate results of their prior publication that these strains can be rescued with riboflavin supplementation and genetic complementation. The authors also highlight ribH gene redundancy in Msm. The highest impact findings of the manuscript are that (1) deletion of Msm and Mtb genes upstream of the canonical activating MR1 precursor ligand, 5-A-RU, suppresses but does not abrogate MAIT cell IFNg responses and (2) deletion of ribH in Mtb that lies downstream of 5-A-RU, leads to enhanced MAIT cell IFNg responses in a previously published select donor biorepository using in vitro infection models. The authors

conclude that 5-A-RU is an essential MAIT cell precursor activating ligand in mycobacteria, paralleling published findings in other bacteria. However, this conclusion was made by inference only, as no direct measurements of metabolite levels were made. We thus recommend either additional experiments or greater caution, as detailed further below. The authors speculate that additional riboflavin-independent MR1 ligands may be generated during Mtb infection to explain why riboflavin auxotrophs remain immunogenic to MAIT cells.

Response: We thank the reviewer for the positive comments. The points made here and in their detailed critique are well taken and are addressed as outlined below.

1) The authors speculate that the abundance of metabolic intermediates 5-A-RU or ribityllumazines that are known to comprise classes of activating MR1 ligands can explain MAIT cell IFNg ELISPOT results, however, no experiments are performed to define these metabolites. Profiling riboflavin metabolite levels in the knockout strains is necessary to make any conclusions about ligands responsible for changes in MAIT cell activation.
Response: While we have added characterization of the known MR1 ligands, we have not defined or synthesized novel ligands in this report. Our prior report (Harriff et al., *Science Immunology* (2018)) demonstrates a broad diversity of MR1 ligands derived from Msm, of which we have only defined lumazine antigens. In ongoing work with the Dobos laboratory, we find that fractionation of Mtb cell wall reveals a broad array of activating fractions, but the characterization of these fractions is an active an incomplete project. As noted, we have speculated that 5-A-RU could lead to a diverse array of ligands, and we stand by this speculation.

2) Insufficient detail is provided about the infection protocols used for the activation assays. Were bacteria used straight from thawed stocks or first cultured, how were CFUs determined to calculate the MOI, and how long did the infection proceed prior to the IFNg release assay? Besides the concern about the metabolic state of just-thawed bacteria, if they were grown with RF supplement, there is presumably also residual intracellular riboflavin that could affect metabolite levels (even if transcript and/or protein levels are unchanged), also depending on the duration of infection (e.g., how long the Mtb were depleted of riboflavin, assuming that there is no access to riboflavin in the host cell). Please provide more detailed and complete methods for the infection to enable better insights into the potential state of the bacteria and resulting presentation/stimulation of host cells.
Response: This has been added to the manuscript, Ln 765-767: "Briefly, a T cell clone is incubated with irradiated PBMC and LCL in addition to anti-CD3 for 12-17 days and frozen. The anti-CD3 is washed out on day 5." Additionally, in Ln 770-770: "D426 and D454 were isolated from a patient that was Mtb-infected (PPD+) and D481 from a patient that had previously had active TB. To generate these clones, CD8+ T cells were sorted from PBMC and incubated in a 96 well limited dilution assay with autologous Mtb-infected dendritic cells for 2 weeks. Wells with growth were screened against autologous and heterologous Mtb-infected dendritic cells. T cell clones that responded only to autologous dendritic cells were characterized to determine the Mtb antigen they recognize (D454 H1-2), and T cell clones that responded to both autologous and heterologous dendritic cells were blocked with various antibodies. D426 G11, D481 C7, and D481 F12 were blocked by anti-MR1

antibody and later found to stain the MR1/5-OP-RU tetramer."

3) Whole genome sequencing methods are cited but there is no mention of these data in the manuscript or as supplemental data. We assume these were used to confirm knockouts and complemented strains, and importantly, to exclude off-target effects. Did the sequencing indeed confirm the genetic manipulation (borders of knockout/insertion and complement insertion) and the absence of secondary mutations compared to the parent strain?
Response: The sequence analysis confirmed the deletions in the respective knockout mutants. We also show the SNP analysis in S10A Table, S10B Tables. The sequencing data has been deposited to http://datadryad.org/share/PHTLPaLIm4vXnHGWrFeeziduU437ye3fixTDZ6GmiwU and are publicly available.

4) Why does the strain Msm ΔribH2 ΔribH1:: ribH2 ribH1 not grow in the presence of RF? It is possible that this strain is compromised at a different location in the genome (see above) and affecting another essential pathway. This also affects the conclusions drawn from figure 5 on MAIT cell activation by Msm.
Response: We thank the reviewer for raising this point. As shown in the new CFU count phenotyping data in S2 Fig A, the Msm Δ*ribH2* Δ*ribH1*::*ribH2 ribH1* strain is indeed able to grow on solid media both in the presence and absence of riboflavin. However, when assessed in liquid culture (Fig. 2D), this strain showed retarded growth kinetics, which was partially restored in the presence of riboflavin, but did not restore growth to the level of wildtype. qRT-PCR analysis confirmed that the level of *ribH1* transcript was expressed at a lower level in the Msm Δ*ribH2* Δ*ribH1*::*ribH2 ribH1* strain than the level in the parental wildtype, which would explain its growth impairment. Furthermore, WGS analysis revealed only two SNP mutations in Msm Δ*ribH2* Δ*ribH1*::*ribH2 ribH1* which were situated in a sigma-70 family RNA polymerase sigma factor (non-synonymous) and an intergenic region, respectively. The contribution, if any, to the growth phenotype of the complemented double mutant, and the incomplete growth restoration by exogenous riboflavin is unclear. This point, and its impact on the conclusions drawn from Fig 5 on MAIT cell activation by Msm is made in the text, Ln 351-353, "Deletion of *ribH1* and *ribH2* alone or in combination had no impact on recognition of Msm by the three TRAV1-2+ clones, D426-G11, D481-C7 and D481-F12."

5) From figure 2A, it is clear that the growth defect in Msm is only caused by the loss of ribH1. Deletion of ribH2 has no effect on the growth of Msm in the absence of riboflavin. This underscores an alternative interpretation to the author's conclusion of "redundancy;" ribH1 may be the only functional lumazine synthase in Msm.
Response: The growth phenotyping on solid media shown in S2 Fig A confirmed that a Msm Δ*ribH1* mutant is still capable of growth in the absence of riboflavin. The ability of the Msm Δ*ribH1* mutant to grow in the absence of riboflavin supplement, albeit at a markedly slower rate than wildtype was confirmed in liquid culture (as shown Fig 2). This was the first indication that there is an alternate source/s of lumazine synthase activity in Msm that can compensate, albeit partially for the loss of RibH1. The fact that deletion of both *ribH1* and *ribH2* rendered Msm incapable of growth on solid media or in liquid culture in the absence of riboflavin supplement is consistent with the designation of RibH2 as an alternate lumazine synthase in Msm.

6) Expression of other rib genes is variable compared to wild type. For example: ribA2 expression is lower in ΔribG and ΔribH2 and higher in ΔribH1 and ΔribC. Notably, ribH expression in ΔribA2 strain is higher compared to the wild type, attributed to the presence of a vector-borne promoter by the authors. These results confound interpretation of the presumed metabolite perturbations and attempts to functionally validate strains using immune assays. Given the sensitivity of flux in metabolic pathways, target-specific knockouts that preserve the activity of other genes in the pathway become crucial for drawing meaningful conclusions.
Response: These experiments were re-done and we observed that *ribA2* expression in the Msm Δ*ribG*, Msm Δ*ribH2*, Msm Δ*ribH1* and Msm Δ*ribC* is equivalent to wildtype levels. In addition, we developed specific, targeted metabolomic assays for DMRL and riboflavin (multiple reaction monitoring mass spectrometry). The method for this assay was added to the manuscript and the results of these analyses are shown in Fig 6 and S8 Fig.

7) Many of these queries may be obviated by metabolic validation, but the manuscript offers limited insights into how these proteomic perturbations impact riboflavin intermediate chemistry.
Response: We agree with this comment and comment 6, above. We developed a targeted metabolomics assay to specifically measure DMRL (and riboflavin) and added the methods and provide the results as described above.

8) Importantly, the immune assays used are reductionist using select donors also used in prior publications, do not include data from donors with negative responses to assess generalizability, and do not include measures of MR1-dependence.
Response: In this report, we have used well characterized, MR1-dependent T cell clones to assess the impact of alterations in the riboflavin pathway. We can say confidently that they are MR1 restricted in that they bind the MR1/5-OP-RU tetramer, do not respond to MR1 knockout cell lines pulsed with Msm or Mtb, and respond to both canonical and non-canonical MR1 antigens. We do not make conclusions in this report about how different donors might respond to different ligands. In our original characterization of MAIT cells, we have found that both Mtb infected and uninfected donors have robust MAIT cells in PBMC characterized by their cell surface phenotype (CD161/CD26/TRAV1-2) and tetramer staining. Hence, we do not have donors available that lack MAIT cells. We believe that adding the HLA-Ia clone adds to the argument that the results are specific for MAIT cells.

9) In figure 5/6 the authors use a well-established IFNg ELISPOT and MAIT cell clones from specific donors also used in their prior work. Further details regarding the methods are requested—how were DCs generated, were they autologous for each donor? Why were DCs used for Msm and BEAS2B used for Mtb? Was IFNg the only response that can be detected from these MAIT cells? The IFNg readout seems surprising considering MAIT cells require cytokine stimulation to generate significant amounts of IFNg—is this provided by the APC and how many APCs are required to generate this response?
Response: These additional methods have been added to the manuscript. Dendritic cells (DCs) were made from a DC donor that matches the D454 H1-2 T cell clone allele (HLA-B*15:01). DCs were used for both Msm and Mtb experiments and that has been updated in the text. These clones express IFNγ and TNF, but do not express IL-17. 1e4 DC and 1e4 T cell clones were added to each well of the ELISPOTs.

Finally, we believe that the response we observe are TCR dependent. Specifically, they respond well to a number of APCs – including BEAS-2b, primary lung epithelial cells, DC, and THP-1s, and require contact with the mycobacterium-infected cell (Gold et. Al, *PLoS Biology* (2010), Harriff et al, *Sci Immunol* (2018), Narayanan et al, *Sci. Rep* (2020), Culicke et al, *Commun. Biol.* (2024), Harriff et al, *PLoS ONE* (2014). Furthermore, we note that these cells do not respond to either BEAS-2b or THP-1 in which MR1 is absent.

10) What other parameters of MAIT cell activation were tested? Was flow cytometry performed? Representative flow cytometry plots of each MAIT cell and dendritic cell clone used in these experiments after thawing of vials from the biorepository should be included with gating strategy and viability. Reference to previous work is not sufficient as distinct vials from the biorepository were thawed for the experiments in this manuscript and variability is to be expected. Are dendritic cells/BEAS the only APCs that are effective for this assay? Representative images of the ELISPOT should be included for the reader to appreciate how the results are calculated.
Response: These MAIT cell clones are expanded using a standardized rapid expansion protocol and tested by ELISPOT after each expansion to confirm that the clones are working as expected. For the data in each figure, the batch of T cell clone was identical as a large stock was expanded, frozen down, confirmed to work, and then the same stock was used for all experiments in that figure. DC are the only APC used (see above). The spot forming units have now been included in S1/S2 Data. For the clones tested, we have previously used a TNF-α capture assay which we found was concordant with the release of IFNγ as measured by ELISPOT. These clones do not express IL-17. As described above, we routinely assess the viability of both our T cell clones and DC.

11) While these donors have previously been used in publications, further details regarding the demographics of the donors including Mtb exposure/IGRA status should be reported.
Response: This has been added to the methods section, Ln 770-774, "D426 and D454 were isolated from a patient that was Mtb-infected (PPD+) and D481 from a patient that had previously had active TB. To generate these clones, CD8+ T cells were sorted from PBMC and incubated in a 96 well limited dilution assay with autologous Mtb-infected dendritic cells for 2 weeks."

12) As the same donors are repeatedly used, questions arise regarding the reproducibility of these results in other donors. Has this assay been performed using MAIT cells from other populations? If the assay is not reproducible in other MAIT cell clones, the negative results should also be displayed. What host factors determine the ability for the MAIT cell clone to respond to Mtb?
Response: We have used T cell clones as a mechanism to evaluate the responses from the mutants of the riboflavin pathway. We do this as it allows us to state with confidence that the responses are MR1-dependent and to include appropriate HLA-Ia controls. In our ongoing work with other collaborators in South Africa, we have derived MR1-dependent clones from both Stellenbosch, UCT, and Durban, and again find that they are MR1-dependent as described above. They also recognized both Msm and Mtb. The topic of TCR diversity, and hence diversity of ligands is one we are very interested in, and the topic of ongoing investigations. Using single cell TCR sequencing, we can state that the vast majority of cells defined by either 5-OP-RU or

Msm tetramer are TRAV1-2 positive. We are certainly interested in evaluating the diversity of ligands in these clinical settings, but this study was not powered to ask what proportion of the responses we have seen might be riboflavin pathway independent.

13) Is prior exposure necessary for or even suppressive of MAIT cell responses during repeat challenge? Are all Mtb-reactive MAIT cells CD8+?
Response: With regards to CD8 – most MAIT cells are either CD8+ or double negative. At least in some cases, we find that CD8 is necessary. Our rapid expansion protocol allows for repeated expansions for both MAIT and non-MAIT T cells, but we cannot say if strategies to induce apoptosis would selectively affect MAIT cells.

14) One TRAV1-2 negative MR1T was used—what proportion of TRAV1-2 negative MAIT cells are Mtb-reactive?
Response: We have very limited experience here. In the peripheral blood, the vast majority of MAIT cells are TRAV1-2 positive, and we have relied on TCR sequencing and cloning to define these cells. We can say that in human cord blood, we see many more TRAV1-2 negative, tetramer positive cells, and cannot comment on how these might respond to Mtb or Msm.

15) The authors reference their previous work on how these MR1T were expanded, however, a brief summary of the methods should be included for completeness including gating strategy for isolation of the original clones (MR1 tetramer vs anti-TRAV1-2?) and method of expansion (antigen vs. mitogen), cryopreservation methods, and details of culturing after thaw prior to performing ELISPOT (eg, can the clones be thawed and used the same day, how many days are they cultured after initial expansion and prior to cryopreservation, or after thaw).
Response: This information has been added to the Methods, under T cell clones, Ln 764-779.

16) No positive control for antigen-specific activation (eg, 5-OP-RU) of these MAIT cell clones are included to confirm reproducibility.
Response: Msm is included as a positive control for antigen-specific activation.

17) No MR1-dependence is measured (eg, anti-MR1). The authors only include mitogen as a positive control.
Response: MR1-dependence has been included for Msm and Mtb, shown in S11 Fig and S13 Fig.

18) Why is the response to Msm mutants measured relative to WT while for Mtb mutants relative to Mtb8.4 T cells? Where is the relative response to Mtb WT?
Response: We do not have an HLA-Ia clone that responds to Msm but also note that we observed more functional variability for the Mtb strains.

19) The manuscript should include the rationale for using different concentrations of riboflavin supplementation in Msm and Mtb.
Response: Done – Ln 524-527: The text has been revised as follows: "Riboflavin auxotrophs were supplemented with exogenous riboflavin at a concentration of 83 µM for Msm or 21 µM for Mtb based on the minimal concentration of riboflavin required

to fully rescue the growth of CRISPRi hypomorphs in genes involved in riboflavin biosynthesis [20].”

20) The layout of the multicolor/pattern bar graphs and multipart legends in Fig 3-5 can be improved for readability.
Response: We agree and have redesigned the figures for clarity.

21) Why was Mtb fbiC knockout not included?
Response: Repeated attempts to create a Mtb ΔfbiC knockout mutant using allelic exchange mutagenesis were unsuccessful, using a suicide plasmid harbouring homologous regions ~1674 bp upstream and 1648 bp downstream in length flanking the deletion leaving 78 amino acid of the FbiC ORF. In this case, no single crossover homologous recombinants were identified from 4 independent electroporations of Mtb using the suicide plasmid. ORBIT-based mutagenesis was not used, however, as an alternate mutagenesis approach for constructing a *fbiC* deletion mutant of Mtb. Given this information, we respectfully submit that doing so would fall beyond the scope of the study. This was added to the text, Ln 532-533: “In contrast, for reasons that are unclear, attempts to generate a knockout mutant of *fbiC* in Mtb were unsuccessful.”

22) Additional discussion is requested as to why ΔribH enhances MAIT cell activation, but not ΔribC when according to the author inference, both should cause accumulation of the activating precursor ligand 5-A-RU.
Response: We speculated that the production of DMRL (dependent on *ribH*) would diminish the availability of 5-AR-U.

23) Line 148: Cited study demonstrates ability to uptake RF, but does not show transport, which generally implies facilitated transport (vs. passive diffusion). Suggest rewording.
Response: Done – Ln 162-164: The text has been revised as follows: “We also demonstrated that Mtb and Msm can assimilate exogenous riboflavin but have yet to establish whether this occurs through passive diffusion or facilitated transport via a non-canonical transporter/s [20].”

24) Line 155: RibA2 catalyzes formation of a substrate used by RibH, which is the enzyme that catalyzes the penultimate step. Suggest rewording.
Response: Done – the text has been revised as follows, Ln 169-171: “Importantly, the deletion mutations in *ribA2* eliminated both GTP cyclohydrolase II and DHBP synthase domains of RibA2; the former catalyzes the first step in the pathway whereas the latter produces a substrate for the RibH enzyme, which catalyzes the penultimate step.”

25) Lines 187- 192: The authors are suggesting that the differences in expression of other rib genes between a knockdown and a knockout can be attributed to the presence of residual protein in the knockdown vs no protein in knockout. This implies some mechanism by which target gene expression would affect other genes, but we are not aware of any evidence to this effect. Suggest rewording or otherwise qualifying this statement accordingly.
Response: This statement is based on work done in *B. subtilis* where it was shown that the riboflavin kinase, RibR, binds the FMN riboswitch (upstream of the riboflavin

biosynthetic operon), regulating the expression of riboflavin biosynthesis. By analogy, we propose that the presence of residual enzyme/s in the hypomorphs may either negatively or positively regulate the expression of other genes in the operon.

26) Line 194: “transcript protein” -> “transcript AND protein”
Response: Done – Ln 239

27) Line 197: Statistical significance analysis of the mean and S.E.M. with two biological replicates is not widely accepted as rigorous for proteomics. Please clarify this analysis.
Response: We repeated all proteomics experiments to include 3 biological replicates and two technical replicates. For figures in supplementary, we have included jitters to ensure all individual data points are visible. We clarified number of samples (biological or technical) for these experiments in the methods section and legend. For the growth kinetics experiments we also repeated to include 4 biological replicates.

28) Figure 4: The most relevant comparison in gene expression and protein abundance among different strains is to the wild type rather than between the complement and +RF condition. Please include the comparison to the wild type statistical analysis of significance.
Response: As above, we clarified this data to improve interpretation. In addition, raw data is provided in the S1 and S2 Data files.

29) Figures 3/5: There is a discrepancy in the smegmatis ribH1/ribH2 KO strains that are characterized for expression/complementation vs. for the immunological assays. In particular the ΔribH1ΔribH2::ribH2 strain in Fig. 5 appears to be used as a ΔribH1 knockout (although this is not explained), but was not validated for RNA/protein expression and so those IFNg release data cannot be interpreted in terms of rib pathway disruption. Suggest that the authors provide fully correspondent data, as is the case for other strains.
Response: We attributed this effect to comparatively weak expression of the complementing genes from nested promoters instead of the main operonic promoter (located upstream of *ribC*). This conclusion was confirmed by qRT-qPCR analysis which revealed that the transcript levels of the complementing ribH1 genes introduced back into the mutant strains were lower than their wildtype levels. The text has been revised to explain this point, Ln 222-231: “Msm Δ*ribH2* Δ*ribH1*::*ribH2 ribH1* showed retarded growth kinetics, which improved to some extent in the presence of riboflavin albeit not to the level of the wildtype. qRT-PCR analysis confirmed that the level of *ribH1* transcript was lower in the Msm Δ*ribH2*Δ*ribH1*::*ribH2 ribH1* strain than in wildtype (**Fig 4A**), which could potentially explain the growth impairment of this complemented strain **(Fig 2F)**. Whole genome sequencing (WGS) revealed only two non-synonymous SNPs in Msm Δ*ribH2* Δ*ribH1*::*ribH2 ribH1* which were situated in a σ^{70} family RNA polymerase sigma factor and an intergenic region, respectively. The contributions thereof (if any) to the growth phenotype of the complemented double mutant and incomplete growth restoration by exogenous riboflavin, are unclear.” In addition, Ln 351-353: “Deletion of *ribH1* and *ribH2* alone or in combination had no impact on recognition of Msm by the three TRAV1-2+ clones, D426-G11, D481-C7 and D481-F12.”
